# Population-specific genetic-risk scores enable improved prediction of mortality within 28 days of sepsis onset: a retrospective Taiwanese cohort study

**DOI:** 10.1186/s40560-025-00783-1

**Published:** 2025-02-26

**Authors:** Ming-Shun Hsieh, Pei-Hsuan Wu, Kuan-Chih Chiu, Shu-Hui Liao, Che-Shao Chen, Tzu-Hung Hsiao, Yi-Ming Chen, Sung-Yuan Hu, Chorng-Kuang How, Amrita Chattopadhyay, Tzu-Pin Lu

**Affiliations:** 1 Department of Emergency Medicine, Taipei Veterans General Hospital, Taoyuan Branch, Taoyuan, 330 Taiwan; 2 Department of Emergency Medicine, Taipei Veterans General Hospital, Taipei, 11217 Taiwan; 3 School of Medicine, National Yang Ming Chiao Tung University, Taipei, 112 Taiwan; 4 Department of Emergency Medicine, Taichung Veterans General Hospital, Taichung, 40705 Taiwan; 5 Institute of Epidemiology and Preventive Medicine, Department of Public Health, National Taiwan University, Taipei, 100 Taiwan; 6 Institute of Environmental and Occupational Health Sciences, College of Public Health, National Taiwan University, Taipei, 100 Taiwan; 7 Department of Pathology and Laboratory, Taipei Veterans General Hospital, Taoyuan Branch, Taoyuan, 330 Taiwan; 8 Department of Medical Research, Taichung Veterans General Hospital, Taichung, Taiwan; 9 Institute of Genomics and Bioinformatics, National Chung Hsing University, Taichung, Taiwan; 10 Research Center for Biomedical Science and Engineering, National Tsing Hua University, Hsinchu, Taiwan; 11 Department of Public Health, Fu Jen Catholic University, New Taipei City, Taiwan; 12 Department of Post-Baccalaureate Medicine, National Chung Hsing University, Taichung, Taiwan; 13 Division of Allergy, Immunology and Rheumatology, Department of Internal Medicine, Taichung Veterans General Hospital, Taichung, Taiwan; 14 School of Medicine, Chung Shan Medical University, Taichung, 40201 Taiwan; 15 Institute of Medicine, Chung Shan Medical University, Taichung, 40201 Taiwan; 16 Department of Post-Baccalaureate Medicine, College of Medicine, National Chung Hsing University, Taichung, 402 Taiwan; 17 Institute of Health Data Analytics and Statistics, Department of Public Health, National Taiwan University, Taipei, 100 Taiwan

**Keywords:** Sepsis, 28-day follow-up, Survival-GWAS, Population-specific variants, Polygenic risk score

## Abstract

**Background:**

Sepsis is characterized by organ dysfunction as a response to infection and is one of the leading causes of mortality and loss of health. The heterogeneous nature of sepsis, along with ethnic differences in susceptibility, challenges a thorough understanding of its etiology. This study aimed to propose prediction models by leveraging genetic-risk scores and clinical variables that can assist in risk stratification of patients.

**Methods:**

A total of 1,403 patients from Taiwan, diagnosed with sepsis, were utilized. Genome-wide survival analysis was conducted, with death within 28 days from sepsis onset, as the primary event to report significantly associated SNPs. A polygenic risk score (PRS-sepsis) was constructed via clumping and thresholding method which was added to clinical-only models to generate better performing prognostic models for identifying high-risk patients. Kaplan–Meier analysis was conducted using PRS-sepsis.

**Results:**

A total of five single-nucleotide-polymorphisms (SNPs) reached genome-wide significance (*p* < 5e-8), and 86 SNPs reached suggestive significance (*p* < 1e-5). The prognostic model using PRS-sepsis showed significantly improved performance with c-index [confidence interval (CI)] of 0.79 [0.62–0.96] and area under receiver operating characteristic curve (AUROC) [CI] of 0.78 [0.75–0.80], in comparison to clinical-only prognostic models (c-index [CI] = 0.63 [0.45– 0.81], AUROC [CI] = 0.61 [0.58–0.64]). The ethnic specificity was established for our proposed models by comparing it with models generated using significant SNPs from prior European studies (c-index [CI] = 0.63 [0.42–0.85], AUROC [CI] = 0.60 [0.58–0.63]). Kaplan–Meier plots showed that patient groups with higher PRSs have inferior survival probability compared to those with lower PRSs.

**Conclusions:**

This study proposed genetic-risk models specific for Taiwanese populations that outperformed clinical-only models. Also it established a strong racial-effect on the underlying genetics of sepsis-related mortality. The model can potentially be used in real clinical setting for deciding precise treatment courses for patients at high-risk thereby reducing the possibility of worse outcomes.

**Supplementary Information:**

The online version contains supplementary material available at 10.1186/s40560-025-00783-1.

## Introduction

Sepsis is characterized by organ dysfunction as a response to host infection and is one of the leading causes of mortality and loss of health [1, 2]. Approximately 11 million people die from sepsis each year, while additional millions are disabled by it [2]. Survivors of sepsis often suffer from long-term physical, cognitive, and psychological deficits [3]. Even though there has been a lot of progress towards early diagnosis, prevention, and treatment of sepsis, the incidence and mortality rates continue to rise [4]. The heterogeneous nature of the condition challenges a thorough understanding of its etiology, leading to bottlenecks in recognizing and treating sepsis [5, 6].

Recent research suggests that the genetic makeup of patients plays a crucial role in their response to sepsis, which could be used to stratify patients who would benefit from certain treatments and targeted therapies [7]. Studies have reported several genes to be associated with various immune and coagulation proteins including interleukins, receptors, and fibrinogen [8]. However, these studies were restricted by smaller patient populations, leading to limited reproducibility. Moreover, most of the important genetic studies were predominantly conducted on the European populations [8–16] and there is relative lack of Asian specific studies. Moreover, there is also a considerable lack of effective genetic prediction models for predicting the risk of mortality due to sepsis, especially among patients of Asian origin. A majority of prior studies focused on candidate regions to identify biomarkers in the form of prognostic genes and variants [9, 14–17]. A recent study by Hernandez‑Beeftink et al*.* included a two-stage genome-wide association study (GWAS) on sepsis patients of European origin to discover variants associated with 28-day mortality [12]. This was the first GWAS performed on patients with sepsis that reported novel associated genetic variants. It is commonly known that the epidemiology of sepsis differs with race, education, geographic location, income, and insurance status [18]. Hence, ethnicity-based analyses are important to understand if the variants and genes identified in European populations are relevant among East Asians, and if not, to identify population-specific single-nucleotide polymorphisms (SNPs) that may influence sepsis-related survival in East Asian patients.

Therefore, this study aims to conduct a genetic association study on a hospital based cohort from Taiwan towards identifying biomarkers associated with mortality due to sepsis within a 28-day period since sepsis incidence. To that end, the study aspired to i) validate prior reported SNPs identified via European studies and ii) identify and report SNPs that are Taiwanese population-specific and not reported among European cohorts. Polygenic risk scores (PRSs) [19] were next calculated using the SNPs identified in this study to establish prognostic models that would potentially allow identification of patients at high risk of mortality from sepsis. These models can facilitate the application of both targeted treatment therapies and preventive strategies to reduce fatal outcomes in patients with sepsis.

## Methods

### Demographic and clinical data

A total of 1,403 patients, diagnosed with sepsis according to the Third International Consensus Definitions for Sepsis and Septic Shock [20], were recruited between January 1, 2017, and December 31, 2021, by the Taichung Veterans General Hospital, Taiwan, and utilized for analysis in this study (**Figure S1**). Demographic information such as the age, sex, height, and weight of each patient, and hospital records such as admission/discharge status, emergency room (ER) admission, transfer to intensive care unit (ICU), discharge or death, and total duration of hospital stay, were obtained. Electronic health records were utilized to access patients’ clinical information including respiratory rate, blood pressure, blood sugar levels, pulse rate, blood parameters, medication usage, medical and emergency procedures, comorbidities, and infection site. Also, information on other comorbidities/complications related to sepsis such as bacteremia, acute renal failure, respiratory failure receiving intubation, stroke, and gastrointestinal bleeding were obtained. All laboratory data were recorded initially on arrival to ER and were followed up after 24 h.

### Scoring systems and immune markers

Sequential Organ Failure Assessment (SOFA) scores were calculated for all patients, which is a cumulative score that allows assessment of the dysfunction of several organs/systems including respiratory, cardiovascular, neurologic, blood, liver, kidney, and blood pressure/hemodynamics in patients with sepsis [21]. The total SOFA score (according to Sepsis-3 definition) is typically calculated upon admission to the ICU and at 24-h intervals thereafter [22]. A higher SOFA score indicates more severe organ dysfunction and is associated with an increased risk of mortality. In this study, the total SOFA score was calculated based on the worst individual-organ-failure score [22]. However, the starting point for this calculation began after the patient was diagnosed with sepsis upon arrival at the ER, continuing for a duration of 24 h thereafter. The Charlson Comorbidity Index (CCI), with weights assigned for all comorbid conditions, was also calculated. Higher CCI scores are indicative of the severity of comorbid conditions, leading to worse prognosis [23].

### Genetic data

All patients included in the study were genotyped using the Taiwan Precision Medicine Initiative (TPMI) chip (Affymetrix Axiom Genomewide TPMI array) containing a total of 684,406 SNPs. In order to obtain the SNPs that were not directly genotyped, genotype imputation was conducted. SHAPEIT2 and IMPUTE2 were used on the quality-checked raw genotype data to obtain whole genome imputed genotypes (93,891,937 SNPs) (Supplementary materials 1). East Asian samples from the 1000 Genomes phase 3 dataset were used as the reference panel [24] for conducting pre-phasing and imputation on each study sample [25].

This study was approved by the ethics committee of Taichung Veterans General Hospital’s institutional review board with IRB # CE16270B-1 and CE23126B. This study falls under the TPMI project, which was initiated in June 2020, is promoted by Academia Sinica in Taiwan, and is executed in 15 hospitals, including Taichung Veterans General Hospital. The genotyping of all study samples was done using customized SNP chips (Axiom Genome-Wide TWB 2.0 Array Plate). All patients provided informed consent.

### Quality control

**Fig. 1:**
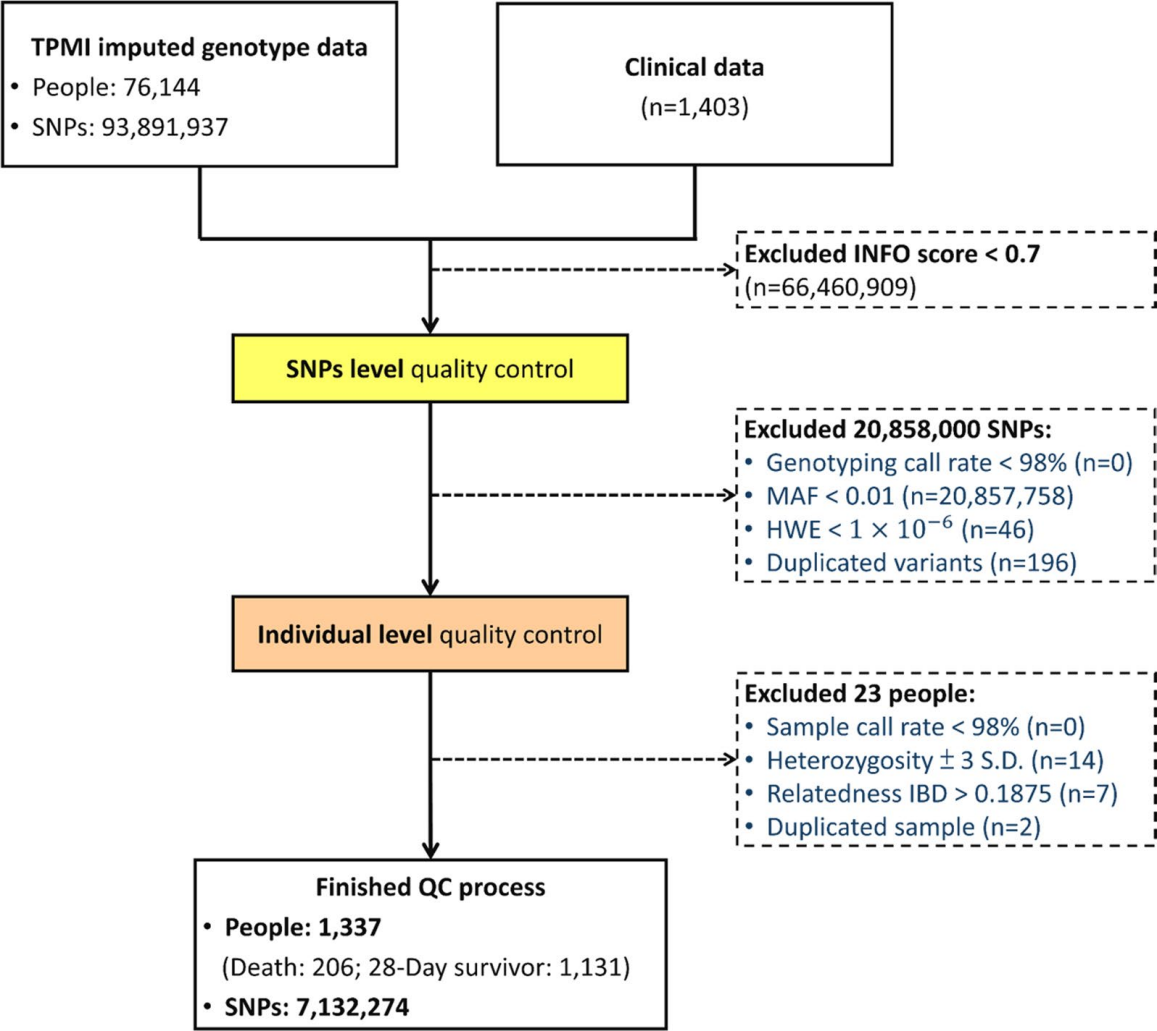
Inclusion and exclusion criteria for study subjects. HWE, Hardy–Weinberg equilibrium; IBD, identity by decent; MAF, minor allele frequency; QC, quality control; SNP, single-nucleotide polymorphism; TPMI, Taiwan Precision Medicine Initiative

SNPs imputed with high accuracy were retained with a threshold of accuracy (INFO score) > 0.7, to be included for analyses (Fig. 1). Quality control measures were applied using PLINK 1.9 [26] and R v4.1.2 [27] to remove poor quality SNPs and individuals [28]. SNPs from mitochondrial DNA or chromosome Y, those with a genotyping call rate < 98%, those deviating from Hardy–Weinberg equilibrium expectations (*p* < 1.0 × 10^–6^), and those with minor allele frequency < 0.01 were excluded (Fig. 1). Samples with missing genotypes (> 2%), with high or low heterozygosity rate ( >|µ ± 3σ|), with sex discrepancies, and with evidence of relatedness (identity by descent > 0.187) were also excluded (Fig. 1). Effects due to population stratification were checked using principal component analysis.

### Genome-wide association analysis of 28-day sepsis survival

Survival genome-wide association study (surv-GWAS), (survival analysis on genome-wide SNP data) adjusted by age, sex, and principal components [29] was performed. Covariates were adjusted to eliminate confounding effects, if any, on the true association signals. The primary endpoint was mortality (event = 1) within a 28-day follow-up period from incidence of sepsis. The subjects were considered without an event if (i) alive at discharge, (ii) the event occurrence after 28 days of follow-up, or (iii) the event was caused due to any cause not sepsis-related. Function *impute2CoxSurv*, from the R package *gwasurvivr v1.16.0* which was specifically developed for imputed genetic data, was implemented [30]. Hazard ratios (HRs) and P-values were used to record the effect size and the associative nature of the SNPs.

### Polygenic risk score (PRS) calculation

Given that the effect size of a single variant is small, PRS was calculated to summarize the genetic burden of an individual’s risk of mortality due to sepsis (PRS-Sepsis) within a 28-day period since its onset. A PRS is the sum of allele counts, weighted by estimated effect sizes as obtained through GWAS. The clumping and thresholding (C + T) technique was utilized to select the SNPs that would be used to construct the PRS. Clumping (C) allows retention of variants that demonstrate weak linkage disequilibrium (LD) within a given genetic distance (500 kb), by iteratively selecting the most significant variant while discarding all the remaining variants that demonstrate a correlation greater than a specified r^2^ value. Thresholding (T), on the other hand, consists of removing variants with a P-value larger than a specified significance level. Clumping thresholds (r^2^) were set at 0.2, 0.4, 0.6, and 0.8, and P-value thresholds were set at 1 × 10^–5^, 1 × 10^–4^, 1 × 10^–3^, 0.01, and 0.05. SNPs selected from all possible combinations of C and T were used to construct PRS. Each of the PRSs were then used to construct baseline models adjusted by age, sex and principle components. A randomly selected 80% of all samples were utilized to train each of the baseline models while the remaining 20% were used as testing set to evaluate each models’ performance via concordance-index (c-index), and P-values. The final PRS-sepsis was selected based on the best testing performance.

### PRS prediction models for 28-day sepsis mortality

Prior study demonstrated that the change of SOFA score in the first 7 days following sepsis onset can predict 28-day mortality with high precision (area under receiver operating curve (AUROC) = 0.85) [31]. Accordingly, the total SOFA score, adjusted for age, and sex, were utilized to construct the “clinical-only” model. The total SOFA score is the sum of the values of six SOFA scores, including platelets (coagulation), Glasgow Coma Scale (neurologic), bilirubin (liver), creatinine (renal), PaO2-FiO2 ratio (respiratory), and mean arterial pressure (cardiovascular). Next, the genetic-risk score, PRS-sepsis, was added to the abovementioned clinical-only model. This was done to test if adding the genetic-risk score would improve the performance of popularly used clinical prediction models. Furthermore, PRS-sepsis was added to a clinical-only-full model constructed out of all variables including total SOFA score, age, sex, body mass index (BMI), norepinephrine usage, septic shock and CCI score, to construct a PRS-sepsis-full model again to evaluate any improvement, if any to the model performance. All models were rigorously compared as explained in the following section to select the final model.

### Comparative analysis with prior models

For comparison purposes, our proposed model using PRS-sepsis were compared with clinical-only models (clinical-only, and clinical-only-full) and prognostic models constructed using PRS-sepsis-Euro (calculated using the significant genetic variants (Table S1) reported in the European studies) as the genetic-risk score predictor. All comparison analyses were conducted using 5-fold cross-validation method. C-index, area under receiver operating characteristics curve (AUROCs), and p-values were reported as metrics for model evaluation based on which the final (best) model was selected. Moreover, the risk that each of the PRS-sepsis scores would confer on the study population were quantified by the hazard rates (HR). Confidence intervals (CI) were provided for both c-index, AUROCs and HRs. Additionally, the model performances were compared using bootstrapping method and DeLong’s test [32].

### Evaluation of the final prediction model

Calibration analysis was conducted for the final model based on a flexible adaptive hazard regression model and smoothed calibration plots for survival outcomes were plotted [3]. Expected and observed survival probabilities were calculated for follow-up intervals of 7 days, 14 days, 21 days, and 28 days, respectively, and were compared with that of the reference values to report P-values were calculated utilizing Namwood–Green–D’Agostino test to confirm that the difference of each of the calibration values with that of the reference values were not significant [33]. Moreover, a decision curve analysis (DCA) was conducted to interpret the clinical benefit of the PRS-sepsis-Final model by visualizing the "net benefit" across a range of patient risk thresholds. It essentially was done to demonstrate how much benefit the model would provide compared to simply treating all or no patients, based on the amount of risk a clinician would allow. The model corresponding to the DCA curve with the highest net benefit across a reasonable range of thresholds would be considered the most clinically useful one. All evaluation analyses were conducted using the testing dataset.

### Kaplan–Meier analysis

Kaplan–Meier analysis was conducted by using PRSs to stratify the patients using different quartiles. PRS-sepsis and PRS-sepsis-Euro, were both grouped according to quartiles and distributions of time to event between the groups were assessed with a log-rank test. The analyses were repeated where both PRS-sepsis and PRS-sepsis-Euro, were divided into five groups based on quintiles. Again, the analyses were conducted on the testing dataset.

## Results

### Patient characteristics

A total of 1,403 sepsis patients from Taiwan with genotype data and clinical information were included in this study (Figure S1). After performing quality control, a total of 1,337 patients with sepsis (206 deaths, 1131 survivors within a 28-day follow-up period) and 7,132,274 SNPs were utilized for further analysis (Fig. 1). Table 1 lists the demographic and clinical characteristics that were reported to be significantly different between patients with and without a mortality event. Older patients (age > 60 years) and males were at a higher risk of mortality within a 28-day follow-up time in comparison to younger and female patients. Interestingly, higher proportions of overweight and obese patients were 28-day survivors in comparison to normal and underweight patients. The mean SOFA, National Early Warning Score 2 (NEWS2), and CCI scores were all significantly higher in patients with an event as opposed to the survivors. More details of all variables in “Descriptive statistics of study populations” in supplementary materials.

**Table 1:** Characteristics associated with 28-day mortality

Characteristics	Missing data	28-day survivor (n = 1,131)	Death (n = 206)	P-value
Age	0 (0)	61.6 (15.3)	71.2 (13.6)	< 0.001
Sex (female), *n (%)*	0 (0)	501 (44.3)	76 (36.9)	0.06
Origin from ED, *n (%)*	0 (0)	969 (85.7)	188 (91.3)	0.04
Length of ED stay, *hour*	0 (0)	20.8 (23.4)	14.0 (13.8)	< 0.001
Septic shock, *n (%)*				
In-hospital	0 (0)	482 (42.6)	146 (70.9)	< 0.001
In-hospital but not ED	0 (0)	371 (32.8)	131 (63.6)	< 0.001
ED	0 (0)	204 (18.0)	60 (29.1)	< 0.001
*Severity*				
CCI score	0 (0)	4.9 (3.4)	7.7 (3.3)	< 0.001
Total SOFA score	0 (0)	7.4 (3.0)	8.3 (3.0)	< 0.001
SOFA score (coagulation)	0 (0)	0.5 (0.9)	0.8 (1.1)	< 0.001
SOFA score (renal)	0 (0)	1.1 (1.3)	1.8 (1.4)	< 0.001
*Laboratory tests*				
NLR	239 (18)	15.8 (20.2)	17.6 (23.2)	< 0.001
*Treatments*				
Norepinephrine usage, *n (%)*				
In-hospital	0 (0)	425 (37.6)	151 (73.3)	< 0.001
In-hospital but not ED	0 (0)	319 (28.2)	130 (63.1)	< 0.001
ED	0 (0)	237 (21.0)	75 (36.4)	< 0.001

ED: emergency department, CCI: Charlson Comorbidity Index, SOFA: Sequential Organ Failure Assessment, NLR: neutrophil-to-lymphocyte ratio. Data are mean (SD) unless otherwise indicated

### Genetic variants associated with 28-day sepsis survival

**Fig. 2:**
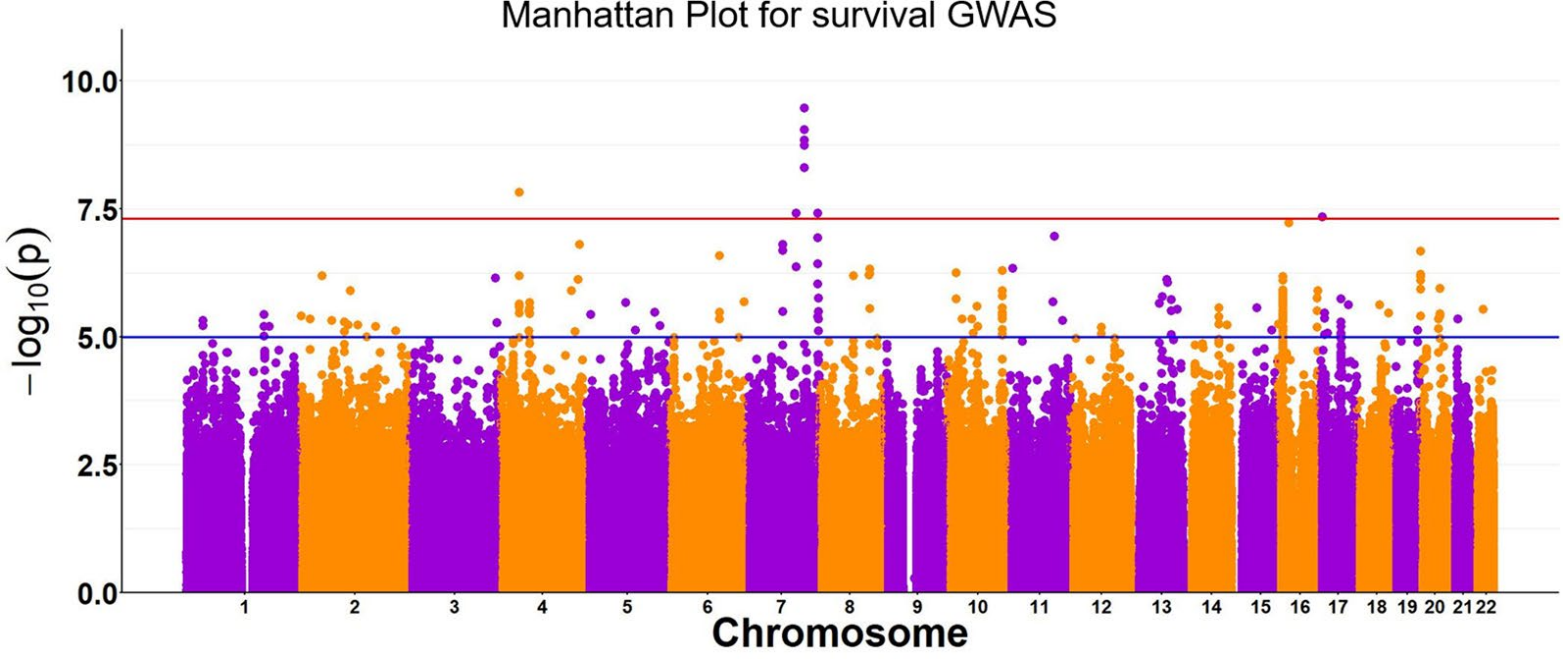
Manhattan plot for genome-wide survival analysis adjusted for age, sex, principal components 1–20, using imputed data with accuracy score, INFO > 0.7. Each data point indicates –log(p-value) for each SNP. The red line is the threshold of p-value for genome-wide significance (5e-8) and the blue line is the threshold for suggestive significance (10^–5^)

Genome-wide survival association tests adjusted by age, sex, and principal components revealed five SNPs (Fig. 2, Table 2) with genome-wide significance (*p* < 5 × 10^–8^). Additionally, 86 SNPs with suggestive significance were also reported via this study (Table S2). The genomic inflation factor (λ) was 0.92, implying no inflation or deviations from the null hypothesis. A further comprehensive search of all the sepsis-associated SNPs from various prior studies was conducted [9, 12, 14–16]. The comparative list is provided in Table S1. All of the prior reported SNPs were identified by our analysis but none reached genome-wide or suggestive significance (*p* < 10^–5^), even though some SNPs (e.g., those from genes *FER, AC003051.1, LINC00887, GAK, CYP11B2, HLA-DPB1, XND1, ADA2,* and *SDHAP1/TFRC*) demonstrated nominal significance (*p* < 0.05).

**Table 2:** SNPs associated with 28-day sepsis survival

								Minor allele frequency					
SNP	Chr	Minor allele	Major allele	MAF	p-value	HR	Gene	TWB	1 KG EAS	1 KG EUR	1 KG AFR	1 KG AMR	1 KG SAS
rs189940971	4	T	C	0.01	1.54 $\times$ 10^–8^	4.86	*ATP8A1*	0.01	0.01	0.002	0.0	0.0	0.0
rs141257999	7	C	T	0.01	3.89 $\times$ 10^–8^	4.45	*DLD*	0.02	0.02	0.0	0.0	0.0	0.0
rs1419466	7	T	C	0.03	3.47 $\times$ 10^–10^	3.84	*GRM8*	0.03	0.04	0.30	0.23	0.30	0.21
rs11769197	7	C	G	0.01	3.89 $\times$ 10^–8^	4.56	*CNPY1*	0.01	0.02	0.09	0.19	0.07	0.04
rs140453155	17	A	G	0.01	4.64 $\times$ 10^–8^	4.53	*PITPNM3*	0.01	0.02	0.0	0.0	0.0	0.0

SNP: single-nucleotide polymorphism, Chr: chromosome number, MAF: minor allele frequency, HR: hazard ratio, TWB: Taiwan Biobank, 1 KG: 1000 genomes, EAS: East Asians, EUR: Europeans, AFR: Africans, AMR: Americans, SAS: South Asians. Each SNP listed in the table was significant at *p* < 5e-8

### Polygenic risk of sepsis and effects on 28‑day sepsis survival

PRS analysis using the C + T method was utilized to calculate PRS-sepsis utilizing population-specific SNPs from this study using the training data. The detailed results of the C + T procedure are provided in Table S3. The best PRS-sepsis (henceforth referred to as PRS-sepsis1) was calculated using 48,546 SNPs, corresponding to an optimal C + T combination of r^2^ > 0.2 and a P-value < 0.05. PRS-sepsis1 was chosen as the best PRS-sepsis score based on a c-index [CI] of 0.82 [0.65–0.98] and AUROC [CI] of 0.78 [0.75–0.81] calculated using the testing data. It is to be noted that to maintain consistency it was ensured that the outcome occurrences were balanced for both the training and the testing cohorts (Tables S4–S5). Furthermore, for comparison purposes, all significant SNPs reported in prior European studies were utilized to calculate a PRS-sepsis-European (PRS-sepsis-Euro) score. Table 3 provides the detailed performance scores of PRS-based models evaluated against clinical-only models utilizing the testing data. Clinical-only model demonstrated a testing c-index [CI] of 0.63 [0.45–0.81] and a testing AUROC [CI] of 0.61 [0.58–0.64] which was better than clinical-only-full model. Hence, PRS-sepsis1 was added to clinical-only model to construct PRS-sepsis-final which showed significantly improved performance (c-index [CI] = 0.79 [0.62–0.96] and AUROC [CI] = 0.78 [0.75–0.80]) when compared to clinical-only model (P = 0.005; Table 4). This model was chosen as the final model as this demonstrated the best performance among all models. Similarly, PRS-sepsis-Euro was added to clinical-only model however the there was no significant improvement (c-index [CI] = 0.63 [0.42–0.85], AUROC [CI] = 0.60 [0.58–0.63]) in the model performance (P = 0.96, Table 4).

**Table 3:** Performance of PRS-based prediction models against clinical-only models

			C-index [95% CI]		Time-dependent AUC [95% CI]
Model	Variables included		Training	Testing	
Clinical-models					
Clinical-only	Total SOFA score, age, sex		0.66 [0.56–0.77]	0.63 [0.45–0.81]	0.61 [0.58–0.64]
Clinical-only-full	Total SOFA score, BMI, Norepinephrine usage in hospital but not in ED, septic shock in hospital. CCI score		0.70 [0.60–0.80]	0.59 [0.36–0.81]	0.58 [0.55–0.62]
PRS-sepsis-model					
PRS-sepsis-final	PRS-sepsis1, total SOFA score, age, sex		**0.80** **[0.72–0.87]**	**0.79** **[0.62–0.96]**	**0.78** **[0.75–0.80]**
PRS-Euro-model					
PRS-sepsis-Euro	PRS-sepsis-Euro, total SOFA score, age, sex		0.80 [0.71–0.87]	0.63 [0.42–0.85]	0.60 [0.58–0.63]

ED: emergency department, CCI: Charlson Comorbidity Index, PRS: polygenic risk score, SOFA: Sequential Organ Failure Assessment; CI: confidence interval. Bold: depicts the PRS-sepsis final model with the best performance, demonstrating highest testing c-index [CI] and highest AUROC [CI] values

**Table 4:** Comparison of model performances

Model	Difference in C-index (95% CI)^#^	P-value
PRS-sepsis-final versus clinical-only model	0.13 (0.05–0.21)	0.005*
PRS-sepsis-final versus clinical-only-full	0.13 (0.03–0.20)	0.01*
PRS-sepsis-Euro versus clinical-only model	0.02 (− 0.07–0.07)	0.96

^#^ Bootstrap method and Delong’s test, ^*^ < 0.05

### Hazards of the PRS-scores among Taiwanese population

To quantify and compare the risk that PRS-sepsis1 and PRS-sepsis-Euro would confer towards sepsis mortality within 28 days of sepsis incidence among Taiwanese patients, the HRs (95% CI) were reported (Table 5). PRS-sepsis1 was found to be a significant predictor of risk of mortality with a HR [CI] = 6.18 [2.60 – 14.68] and P < 0.001. The HR was calculated with PRS = 0 as reference, therefore, for PRS-sepsis1, the risk of mortality increases 6.18 times with per unit increase of the PRS value. Therefore, PRS-sepsis1 was considered a good stratification tool for identifying high risk (28-day mortality) patients in Taiwan. However, PRS-sepsis-Euro was found to have HRs very close to one (HR [CI] = 0.67 [0.47 – 0.97], P = 0.03), rendering it inappropriate for risk stratification of 28-day mortality for patients in Taiwan (Table 5).

**Table 5:** The hazard-ratios of polygenic risk scores for 28-day mortality due to sepsis in Taiwanese patients

	Mortality	
PRS	HR [95% CI]	P-value
PRS-sepsis1	6.18 [2.60–14.68]	< 0.001*
PRS-sepsis-Euro	0.67 [0.47–0.97]	0.03*

CI: confidence interval, HR: hazard ratio, PRS: polygenic risk score

* p-value < 0.05

### Calibration analysis

**Fig. 3:**
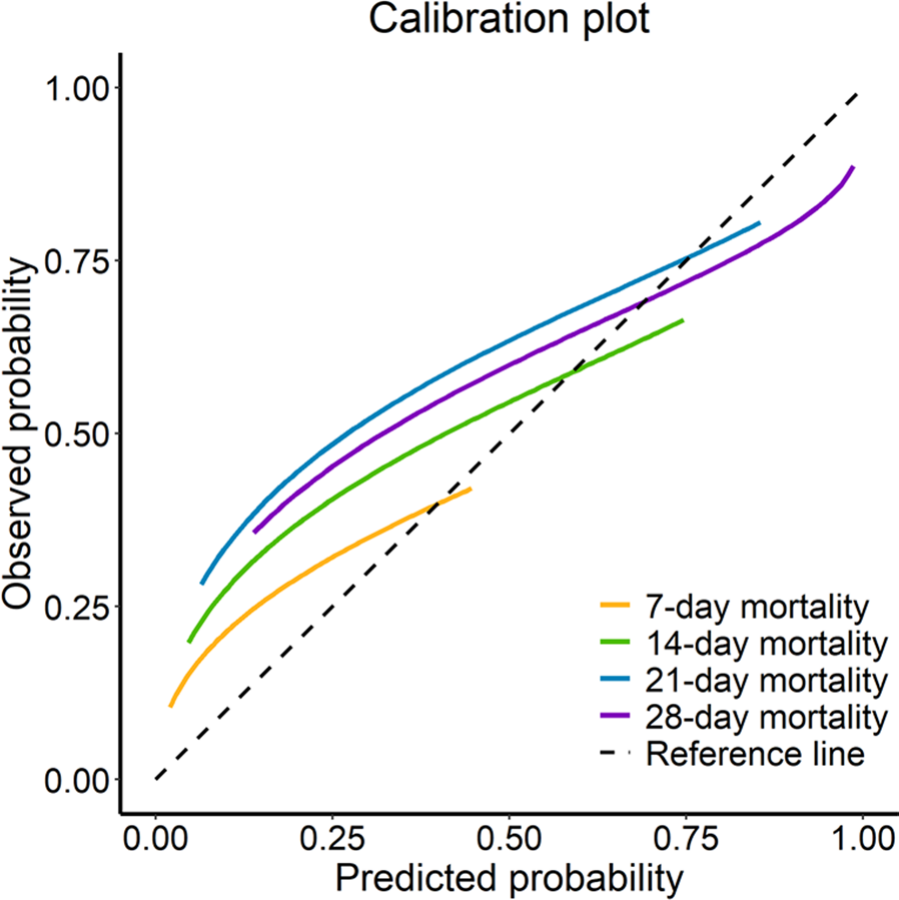
Calibration plot for PRS-sepsis-Final model. Calibrations conducted across different risk levels (x-axis) for follow-up durations of 7 day (orange), 14 days (green), 21 days (blue), and 28 days (purple), respectively. The dashed black line represents the reference line

Calibration analyses were conducted across different risk levels to predict mortality within 7 days, 14 days, 21 days and 28 days, respectively, since sepsis onset, to judge if the PRS-sepsis-Final model was well calibrated or not. The calibration plot demonstrated approximate convergence of the expected and observed probability for high-risk patients where all the lines intersected the reference (dotted) line (Fig. 3). Namwood–Green–D’Agostino test demonstrated that the differences of each of the 7-day, 14-day, 21-day, and 28-day mortality from the reference values were not significant with p-values of 0.79, 0.83, 0.26, and 0.09, respectively. This implied that the model’s predictions were consistently in agreement with the true survival outcomes, thereby indicating that the model demonstrated good predictive performance and the ability to provide reliable risk estimations.

### Decision curve analysis

**Fig. 4:**
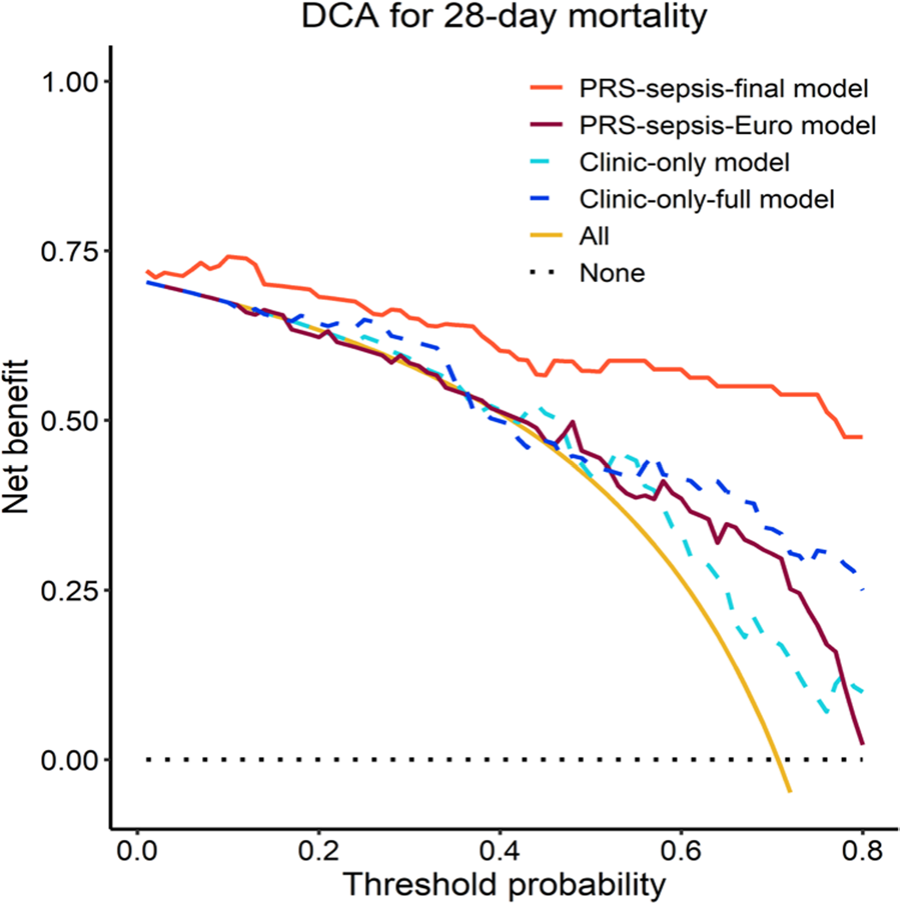
Decision curve analysis plot representing the "net benefit" of the PRS-sepsis-Final against PRS-sepsis-Euro, clinical-only and clinical-only-full models, across a range of patient risk thresholds, to demonstrate how much benefit the model would provide compared to simply treating all or no patients X-axis: threshold probabilities. Y-axis: net benefit probabilities. Red: PRS-sepsis-final model, brown: PRS-sepsis-Euro model, light blue (dashed): clinical-only model, dark blue (dashed): clinical-only-full model, orange: all treatment, black line (dotted): no treatment

Figure 4 demonstrates the net benefit of PRS-sepsis-Final model against PRS-sepsis-Euro, and clinical-only models when compared with 'treating all' or 'treating no patients'. As can be observed, the PRS-sepsis-Final demonstrates the highest net-benefit in comparison to all other models implying that PRS-sepsis-Final is the most clinically useful one.

### Kaplan–Meier analysis

**Fig. 5:**
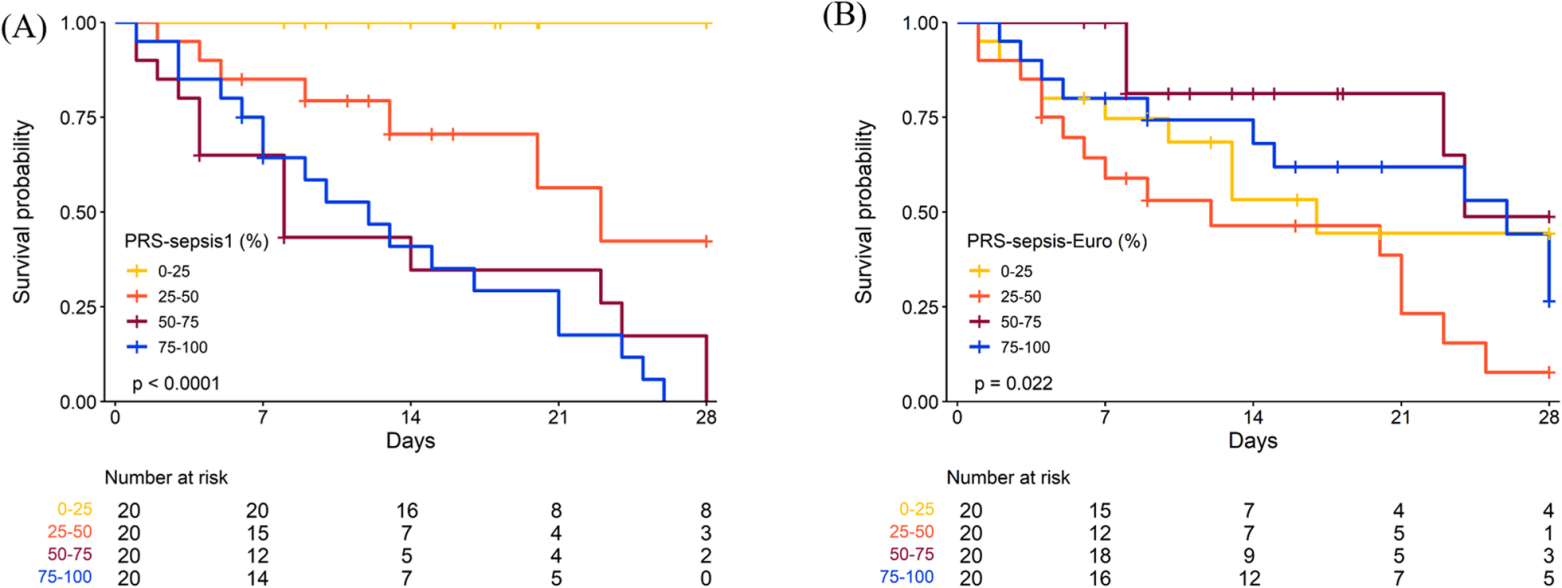
Kaplan–Meier plots showing the survival probability of sepsis patients using testing dataset (80 patients) stratified by PRS quartiles (yellow: PRS 0th–25th percentile; orange: PRS 25th–50th percentile; brown: PRS 50th–75th percentile; blue: PRS 75th—100th percentile.** A** Kaplan–Meier plots stratified by PRS-sepsis1 quartiles.** B** Kaplan–Meier plots stratified by PRS-sepsis-Euro quartiles. Log rank tests were conducted to provide p-values

Kaplan–Meier analysis was conducted by using PRSs to stratify the patients into different quartiles and quintiles of the PRS-sepsis1 and PRS-sepsis-Euro, and distributions of time to event between the groups were assessed with a log-rank test. Kaplan–Meier plots using quartiles (Fig. 5) and quintiles (Figure S1) were then generated using PRS-sepsis1 (Fig. 5A, Figure S1A), PRS-sepsis-Euro, (Fig. 5B, Figure S1B), respectively. The results demonstrated that patient groups with higher-PRS quartile and quintiles using PRS-sepsis1 had significantly inferior survival probability than those with low-PRS quartiles and quintiles, respectively (*p* < 0.001), while using PRS-sepsis-Euro, the trend was quite different, where, Taiwanese subjects with lower PRS-sepsis-Euro, demonstrated worse survival than those with higher PRS-sepsis-Euro. The above, therefore, confirmed the existence of strong racial effect on the underlying genetics of sepsis-related mortality.

## Discussion

Sepsis is not a standalone disease but a syndrome with various symptoms due an over-activation of the host’s immune responses caused by pathogens and host factors, which if not promptly diagnosed and treated can lead to death [34]. This study identified genetic variants which are significantly associated with risk of sepsis mortality in Taiwanese patients and utilized a genetic-risk score (PRS-sepsis1) to propose prognostic models for identifying patients that are potentially at a higher risk of mortality within a 28-day period since sepsis onset. The findings indicated that adding genetic information to clinical-only models improved the models’ performance substantially. In addition, most of the GWASs on sepsis to date have been focused primarily on European populations, and the current study is one of the first to verify if significant associations among Caucasians were also significant among Asian patients. In our results, there was no overlap of significant SNPs from Taiwanese patients with those from prior studies, and five novel SNPs with genome-wide significance (*p* < 5 × 10^–8^) were identified as Taiwanese population-specific SNPs. Sepsis has been found to act differently in people with different ethnic origins [35]. Additionally, socioeconomic factors such as education, income, health insurance, place of residence, and occupation have all been shown to be potential barriers to accessing healthcare, leading to higher mortality due to sepsis [35]. In this study the HRs and Kaplan–Meier analysis using the PRS-sepsis1 and PRS-sepsis-Euro both reiterated the strong racial effect on the underlying genetics that drives risk of sepsis mortality. Therefore, prediction models using PRSs from our study will be clinically useful as they will enable early screening of patients from Taiwan who are at a higher risk of mortality due to sepsis.

Over the past two decades, a multitude of experimental studies and clinical trials have demonstrated that sepsis occurs via the host’s immune response to pathogens triggering multi-organ failure, the process of which is initiated via the activation of innate immune cells such as macrophages, monocytes, neutrophils, and natural killer cells, followed by various intermediate processes, which eventually allows production of crucial cytokines and caspases that enable programmed cell death [36, 37]. Early recognition of sepsis and early management has been shown to reduce mortality. Sepsis has a strong genetic component (~ 10%) and individuals demonstrates a fivefold higher risk of mortality due to infection if a biologic parent died of it [38]. Moreover, sepsis-related mortality has a higher heritability than cardiovascular diseases or cancer [39]. All of the above indicates the importance of identifying appropriate genetic markers that can help mitigate adverse outcomes. SNP rs189940971 reported via this study was found in gene *ATP8A1*, which is expressed at higher levels in sepsis patients [40] and involved in several functions related to lipid transport. Gene ontology annotations of the *DLD* gene containing SNP rs141257999, found to be associated in this study, revealed its involvement in oxidoreductase activity and associations with dihydrolipoamide dehydrogenase deficiency and methemoglobinemia due to deficiency of methemoglobin reductase. However, no prior records exist as to its association with immunity related to sepsis. SNP, rs1419466, was found in gene *GRM8*, which has been reported to play a protective role against inflammation of the central nervous system. SNP rs11769197 was mapped to *CNPY1*, which has been reported to have associations with autism, schizophrenia, urate measurement, COVID-19, and bone density. SNP rs140453155 was found in gene *PITPNM3*, which is known to be associated with blood pressure. All of the latter three SNPs were novel in this study, with no prior reported association with sepsis. A further functional analysis of the genes *ATP8A1*, *DLD*, *GRM8*, *CNPY1*, and *PITPNM3* (Table 2) using Functional Mapping and Annotation (FUMA) demonstrated the top five tissues with higher differential expression as brain, esophagus, breast, ovary, and adipose tissue, specifically the caudate basal ganglia, the gastroesophageal junction, mammary tissue, and the esophageal muscularis (Figures S2– S4). Gene *ATP8A1* was found to be highly differentially expressed in brain, and *CNPY1* was found to be strongly differentially expressed in the cerebellum. These results are consistent with the previous finding that sepsis leads to cerebral dysfunction and may cause blood–brain barrier disruption, neuroinflammation, and hypoperfusion that induces long- or short-term cognitive impairment [41]. *DLD* was found to be highly expressed in lymphocytes, and *PITPNM3* was overexpressed in spleen. Disruption of immune system cells plays a crucial role in inducing abnormal levels of immunoregulatory molecules, and these abnormalities observed in circulating lymphocytes are key to understanding the pathophysiology of sepsis [42].

Sepsis is life threatening due to its heterogeneous nature, which is a result of complex signaling pathways and a poorly regulated host response. Recognizing patients that are at a higher risk of mortality, early on, can lead to better interventions that may reduce the severity of the consequences of sepsis. Towards that end, this study utilized the underlying risk variants associated with sepsis in Taiwanese patients to develop a genetic-risk score and included it along with clinical data to generate various prognostic models that can assist in identifying patients with higher risk of mortality due to sepsis within a 28-day follow-up period. PRS-sepsis-final model with risk score, PRS-sepsis1, performed with significantly better discrimination and AUROC than clinical-only and clinical-only-full models. This was not surprising given the strong genetic component that underlies the risk of mortality due to sepsis [38, 39]. The efficacy of sepsis treatment is low and therefore frequently leads to severe consequences, including stroke and extended hemodialysis. The PRS-sepsis-final model signifies a preliminary advancement in integrating genetic elements into clinical practice, offering a benchmark for clinicians in their decision-making processes. The model can have potential contribution in real clinical setting for decision-making, by facilitating prompt and thorough treatment planning for patients at high risk. The model also would provide physicians with scientifically valid rationale to take into consideration the genetic constitution of a specific patient while designing patient specific precise treatment that would contribute in reducing worse outcomes. PRS-sepsis models utilizing PRS-sepsis2 (C + T: *r*^2^ = 0.4, *p* = 1e-4) and PRS-sepsis3 (C + T: *r*^2^ = 0.8, *p* = 1e-5), utilizing 407 and 95 SNPs, respectively (Table S3), could potentially be alternate candidate genetic-risk scores for constructing Taiwan-specific sepsis models. Analyses were done using both PRS-sepsis2 and PRS-sepsis3 by adding them to clinical-only models. Even though PRS-sepsis-final performed the best among all, models with PRS-sepsis2 and PRS-sepsis3 were close second and third, respectively (results not shown). It is to be noted that models with PRS-sepsis2 and PRS-sepsis3 could potentially be more practicable in real clinical settings due to the lower number of SNP markers required to be genotyped for PRS calculation.

The traditional explanation of the effect that ethnic differences confer upon incidence and prevalence of complex diseases has mostly focused on environmental, social, cultural, or economic factors; however, the substantial role that genetics confer is often not taken into consideration. Large numbers of genetic markers associated with inflammatory and immunologic diseases have been observed with significant frequency differences among ethnically distinct populations [43, 44]. Therefore, connecting genetic profiles with the ethnicity of individuals bridges race and genetics and demonstrates the differences in high-risk alleles that exist in various ethnic groups. This was further elucidated for sepsis via this study, as the variants reported to be significant in European patients failed to show validity in Taiwanese patients. Moreover, PRS-based models utilizing population-specific SNPs identified in this study were found to perform better in Taiwanese populations than models derived from European populations (PRS-sepsis-Euro, calculated using risk variants reported by Hernandez‑Beeftink et al*.* and other prior studies [9, 12, 14–16]).

One of the limitations of this study was the lack of a prospective external cohort to conduct validation of the performance of the prognostic models; however, extensive internal validation and comparison analyses were conducted instead. Moreover, in addition to genetic-risk factors, we ensured that all models incorporated a broad set of clinical factors that could potentially have association with sepsis, and thus we believe that the findings could be generalized to other larger patient populations. Nevertheless, future studies should be conducted to validate the findings using independent cohorts of patients.

## Conclusion

This study presents a comprehensive analysis on patients from Taiwan and proposes precise and high-performing genetic-risk score-based model that would allow early identification of patients who are at higher risk of fatal outcomes due to sepsis within a 28-day follow-up period, leading to better disease management protocols. Our findings provide evidence that adding genetic-risk scores to prognostic models increases their predictive ability and that there is a strong racial effect on the underlying genetics of sepsis-related mortality. The model can potentially be used in real clinical setting for deciding precise treatment course for patients at high-risk, thereby reducing the possibility of worse outcomes.

## Supplementary Information

Supplementary material 1: Genotype-imputation. Descriptive statistics of study populations

Supplementary material 2: Figure S1. Exclusion criteria and study samples included in the study. Figure S2. Heatmap showing tissue based differential expression analysis using FUMA for genes mapped from genome-wide significant SNPs. Figure S3. General tissue specific up-regulated and down-regulated genes mapped from genome-wide significant SNPs via FUMA. Figure S4. Detailed tissue specific up-regulated and down-regulated genes mapped from genome-wide significant SNPs via FUMA. Table S1. SNPs found previously to be associated with sepsis susceptibility in genome-wide association studies. Table S2. List of suggestively significant SNPs (p<1 x10-5). Table S3. The performance of the polygenic risk score in the Cox prediction model using clumping and thresholding method (C+T) parameters. Table S4. Characteristics associated with 28-day mortality in the training dataset. Table S5. Characteristics associated with 28-day mortality in the testing dataset.

## Data Availability

The data that support the findings of this study are available from [Taichung Veterans General Hospital] but restrictions apply to the availability of these data, which were used under license for the current study, and so are not publicly available. Data are, however, available from the authors upon reasonable request and with permission of [Taichung Veterans General Hospital].

## Abbreviations

GWAS: Genome-wide association study
PRS: Polygenic risk scores
SOFA: Sequential Organ Failure Assessment
CCI: Charlson Comorbidity Index
CRP: C-reactive protein
NLR: Neutrophil-to-lymphocyte ratio
SNP: Single-nucleotide polymorphism
HR: Hazard ratio
C + T: Clumping and thresholding
LD: Linkage disequilibrium
NEWS2: National Early Warning Score 2
DCA: Decision curve analysis
