# Supplementary material for: Population-specific genetic-risk scores enable improved prediction of mortality within 28 days of sepsis onset: a retrospective Taiwanese cohort study
Source: J Intensive Care. 2025 Feb 26;13:11. doi: 10.1186/s40560-025-00783-1 (PMC11863615; doi:10.1186/s40560-025-00783-1)
Supplement: Supplementary file 1 — Supplementary material 1: Genotype-imputation. Descriptive statistics of study populations [file 40560_2025_783_MOESM1_ESM.docx]

**Supplementary materials**

**Population-specific genetic risk scores enable improved prediction of mortality within 28 days of sepsis onset: a retrospective Taiwanese cohort study**

Ming-Shun Hsieh^1,2,3,4^, Pei-Hsuan Wu^5^, Kuan-Chih Chiu^6^, Shu-Hui Liao^7^, Che-Shao Chen^1^, Tzu-Hung Hsiao^8,9,10,11^, Yi-Ming Chen^8,12,13^, Sung-Yuan Hu^4,14,15,16^, Chorng-Kuang How^2,3^, Amrita Chattopadhyay^5,^*, Tzu-Pin Lu^17,^*

^1^Department of Emergency Medicine, Taipei Veterans General Hospital, Taoyuan Branch, Taoyuan 330, Taiwan

^2^Department of Emergency Medicine, Taipei Veterans General Hospital, Taipei 11217, Taiwan

^3^School of Medicine, National Yang Ming Chiao Tung University, Taipei 112, Taiwan

^4^Department of Emergency Medicine, Taichung Veterans General Hospital, Taichung 40705, Taiwan

^5^Institute of Epidemiology and Preventive Medicine, Department of Public Health, National Taiwan University, Taipei 100, Taiwan

^6^Institute of Environmental and Occupational Health Sciences, College of Public Health, National Taiwan University, Taipei 100, Taiwan

^7^Department of Pathology and Laboratory, Taipei Veterans General Hospital, Taoyuan Branch, Taoyuan 330, Taiwan

^8^Department of Medical Research, Taichung Veterans General Hospital, Taichung, Taiwan

^9^Institute of Genomics and Bioinformatics, National Chung Hsing University, Taichung, Taiwan

^10^Research Center for Biomedical Science and Engineering, National Tsing Hua University, Hsinchu, Taiwan

^11^Department of Public Health, Fu Jen Catholic University, New Taipei City, Taiwan

^12^Department of Post-Baccalaureate Medicine, National Chung Hsing University, Taichung, Taiwan

^13^Division of Allergy, Immunology and Rheumatology, Department of Internal Medicine, Taichung Veterans General Hospital, Taichung, Taiwan

^14^School of Medicine, Chung Shan Medical University, Taichung 40201, Taiwan

^15^Institute of Medicine, Chung Shan Medical University, Taichung 40201, Taiwan

^16^Department of Post-Baccalaureate Medicine, College of Medicine, National Chung Hsing University, Taichung 402, Taiwan

^17^Institute of Health Data Analytics and Statistics, Department of Public Health, National Taiwan University, Taipei 100, Taiwan

* Corresponding Authors:

**1. Amrita Chattopadhyay**

Institute of Epidemiology and Preventive Medicine, Department of Public Health, National Taiwan University, Taipei 10055, Taiwan

Phone: +886-2-3366-8265, Email: amrita@ntu.edu.tw

**2. Tzu-Pin Lu**

Institute of Health Data Analytics and Statistics, Department of Public Health, National Taiwan University, Taipei 10055, Taiwan

Phone: +886-2-3366-8042, Fax: +886-2-3322-4179, E-mail: tplu@ntu.edu.tw

**Genotype- imputation**

Raw microarray SNP data genotyped using Taiwan Precision Medicine Initiative (TPMI) chip (Affymetrix Axiom Genomewide TPMI array) containing a total of 684,406 SNPs, were analyzed through a genotype imputation pipeline for obtaining a total of 93,891,937 imputed SNPs. The SNPs and individuals were first passed through quality control, to achieve high-quality imputation results, by excluding both low quality data. Plink1.9 (1) were utilized to exclude SNPs with call-rates <95% and those which deviated from Hardy-Weinberg equilibrium with P-value < 0.000001and samples with missing rates >95%. Also, very rare SNPs were removed (MAF <0.01%). The remaining SNPs were pre-phased using SHAPEIT2 (2) and imputed using IMPUTE2 (3) by utilizing the reference panel 1000 genomes phase III, East Asian samples (4).

**Descriptive statistics of study populations**

A total of 1,403 sepsis patients from Taiwan with genotype data and clinical information were included in this study. After performing quality control, a total of 1,337 patients with sepsis (206 deaths, 1131 survivors within a 28-day follow up period) and 7,132,274 SNPs were utilized for further analysis (**Figure 1**). **Table 1** lists the demographic and clinical characteristics that were reported to be significantly different between patients with and without a mortality event. Older patients (age >60 years) and males were at a higher risk of mortality within a 28-day follow-up time in comparison to younger and female patients. Interestingly, higher proportions of overweight and obese patients were 28-day survivors in comparison to normal and underweight patients. The mean SOFA, National Early Warning Score 2 (NEWS2), and CCI scores were all significantly higher in patients with an event as opposed to the survivors. Laboratory tests demonstrated significantly lower mean levels of platelets, lymphocytes, albumin and HbA1c in patients with events, while other levels, including creatinine, and bilirubin, were higher in patients with events. Lactic acidosis is observed often in patients with septic shock because of tissue hypoperfusion, hence lactate levels were significantly higher in the mortality group. Procalcitonin levels were also observed to be significantly higher in the mortality group, as procalcitonin is a marker for infection that is most often enhanced in septic shock. Medications such as norepinephrine were administered significantly more to patients with an event than those with no event within the 28-day follow-up period. Infection in the respiratory system was significantly more frequent while that in the genitourinary system was significantly less frequent in patients who died due to sepsis within the follow-up period. Device infection and infection at other sites were also significantly lower in patients with an event in comparison to survivors.

References

1. Chang CC, Chow CC, Tellier LC, Vattikuti S, Purcell SM, Lee JJ. Second-generation PLINK: rising to the challenge of larger and richer datasets. Gigascience. 2015;4(1):s13742-015-0047-8.

2. Delaneau O, Zagury J-F, Marchini J. Improved whole-chromosome phasing for disease and population genetic studies. Nature methods. 2013;10(1):5-6.

3. Howie BN, Donnelly P, Marchini J. A flexible and accurate genotype imputation method for the next generation of genome-wide association studies. PLoS genetics. 2009;5(6):e1000529.

4. Consortium GP. A global reference for human genetic variation. Nature. 2015;526(7571):68.
