# Supplementary material for: Population-specific genetic-risk scores enable improved prediction of mortality within 28 days of sepsis onset: a retrospective Taiwanese cohort study
Source: J Intensive Care. 2025 Feb 26;13:11. doi: 10.1186/s40560-025-00783-1 (PMC11863615; doi:10.1186/s40560-025-00783-1)
Supplement: Supplementary file 2 — Supplementary material 2: Figure S1. Exclusion criteria and study samples included in the study. Figure S2. Heatmap showing tissue based differential expression analysis using FUMA for genes mapped from genome-wide significant SNPs. Figure S3. General tissue specific up-regulated and down-regulated genes mapped from genome-wide significant SNPs via FUMA. Figure S4. Detailed tissue specific up-regulated and down-regulated genes mapped from genome-wide significant SNPs via FUMA. Table S1. SNPs found previously to be associated with sepsis susceptibility in genome-wide association studies. Table S2. List of suggestively significant SNPs (p<1 x10^-5^). Table S3. The performance of the polygenic risk score in the Cox prediction model using clumping and thresholding method (C+T) parameters. Table S4. Characteristics associated with 28-day mortality in the training dataset. Table S5. Characteristics associated with 28-day mortality in the testing dataset. [file 40560_2025_783_MOESM2_ESM.docx]

**Supplementary files**

**Population-specific genetic risk scores enable improved prediction of mortality within 28 days of sepsis onset: a retrospective Taiwanese cohort study**

Ming-Shun Hsieh^1,2,3,4^, Pei-Hsuan Wu^5^, Kuan-Chih Chiu^6^, Shu-Hui Liao^7^, Che-Shao Chen^1^, Tzu-Hung Hsiao^8,9,10,11^, Yi-Ming Chen^8,12,13^, Sung-Yuan Hu^4,14,15,16^, Chorng-Kuang How^2,3^, Amrita Chattopadhyay^5,*^, Tzu-Pin Lu^17,*^

^1^Department of Emergency Medicine, Taipei Veterans General Hospital, Taoyuan Branch, Taoyuan 330, Taiwan

^2^Department of Emergency Medicine, Taipei Veterans General Hospital, Taipei 11217, Taiwan

^3^School of Medicine, National Yang Ming Chiao Tung University, Taipei 112, Taiwan

^4^Department of Emergency Medicine, Taichung Veterans General Hospital, Taichung 40705, Taiwan

^5^Institute of Epidemiology and Preventive Medicine, Department of Public Health, National Taiwan University, Taipei 100, Taiwan

^6^Institute of Environmental and Occupational Health Sciences, College of Public Health, National Taiwan University, Taipei 100, Taiwan

^7^Department of Pathology and Laboratory, Taipei Veterans General Hospital, Taoyuan Branch, Taoyuan 330, Taiwan

^8^Department of Medical Research, Taichung Veterans General Hospital, Taichung, Taiwan

^9^Institute of Genomics and Bioinformatics, National Chung Hsing University, Taichung, Taiwan

^10^Research Center for Biomedical Science and Engineering, National Tsing Hua University, Hsinchu, Taiwan

^11^Department of Public Health, Fu Jen Catholic University, New Taipei City, Taiwan

^12^Department of Post-Baccalaureate Medicine, National Chung Hsing University, Taichung, Taiwan

^13^Division of Allergy, Immunology and Rheumatology, Department of Internal Medicine, Taichung Veterans General Hospital, Taichung, Taiwan

^14^School of Medicine, Chung Shan Medical University, Taichung 40201, Taiwan

^15^Institute of Medicine, Chung Shan Medical University, Taichung 40201, Taiwan

^16^Department of Post-Baccalaureate Medicine, College of Medicine, National Chung Hsing University, Taichung 402, Taiwan

^17^Institute of Health Data Analytics and Statistics, Department of Public Health, National Taiwan University, Taipei 100, Taiwan

* Corresponding authors

1. Amrita Chattopadhyay

Institute of Epidemiology and Preventive Medicine, Department of Public Health, National Taiwan University, Taipei 10055, Taiwan

Phone: +886-2-3366-8265, Email: amrita@ntu.edu.tw

2. Tzu-Pin Lu

Institute of Health Data Analytics and Statistics, Department of Public Health, National Taiwan University, Taipei 10055, Taiwan

Phone: +886-2-3366-8042, Fax: +886-2-3322-4179, E-mail: tplu@ntu.edu.tw

1. **Supplementary figures**


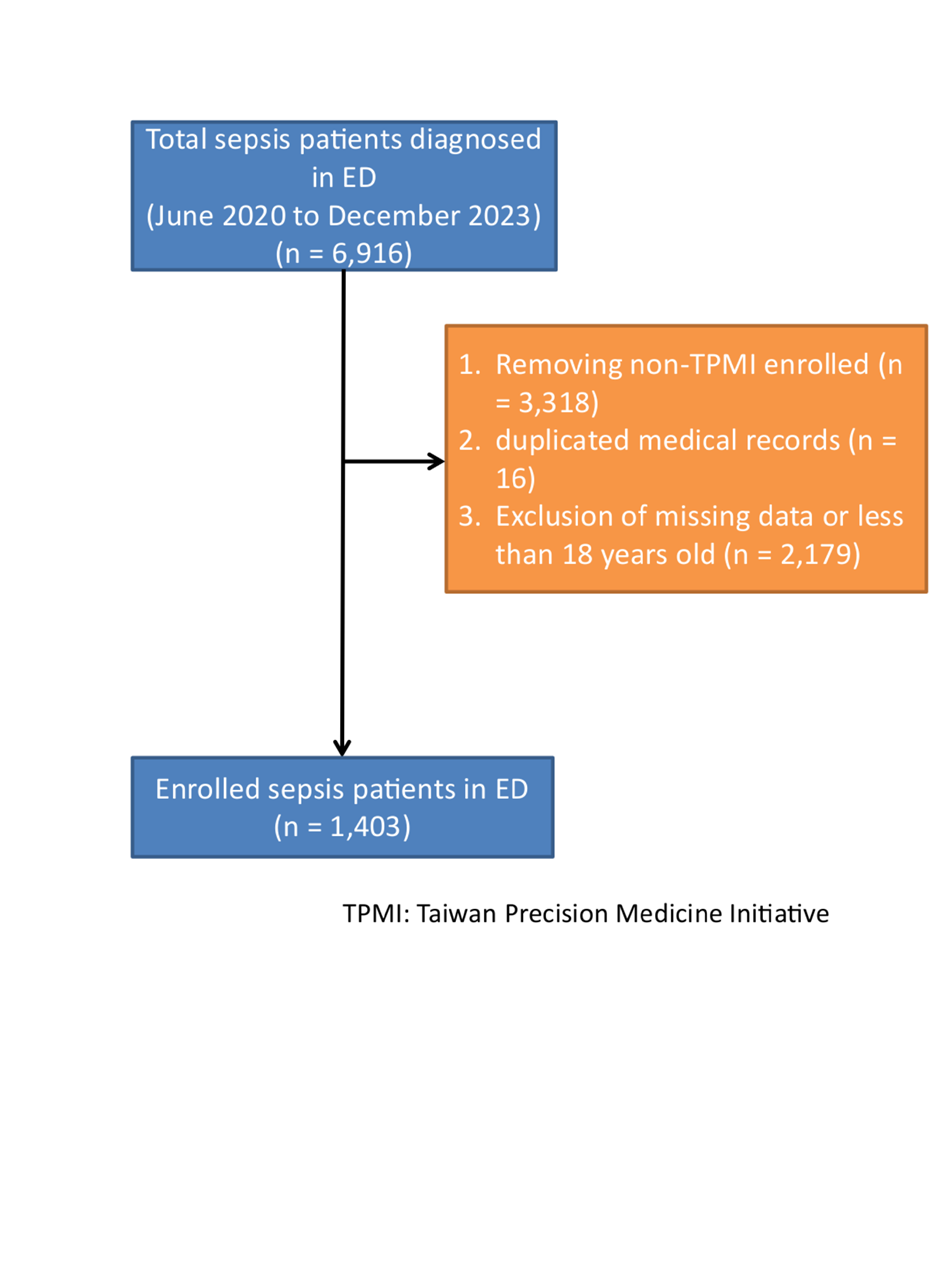


**Figure S1. Exclusion criteria and study samples included in the study**


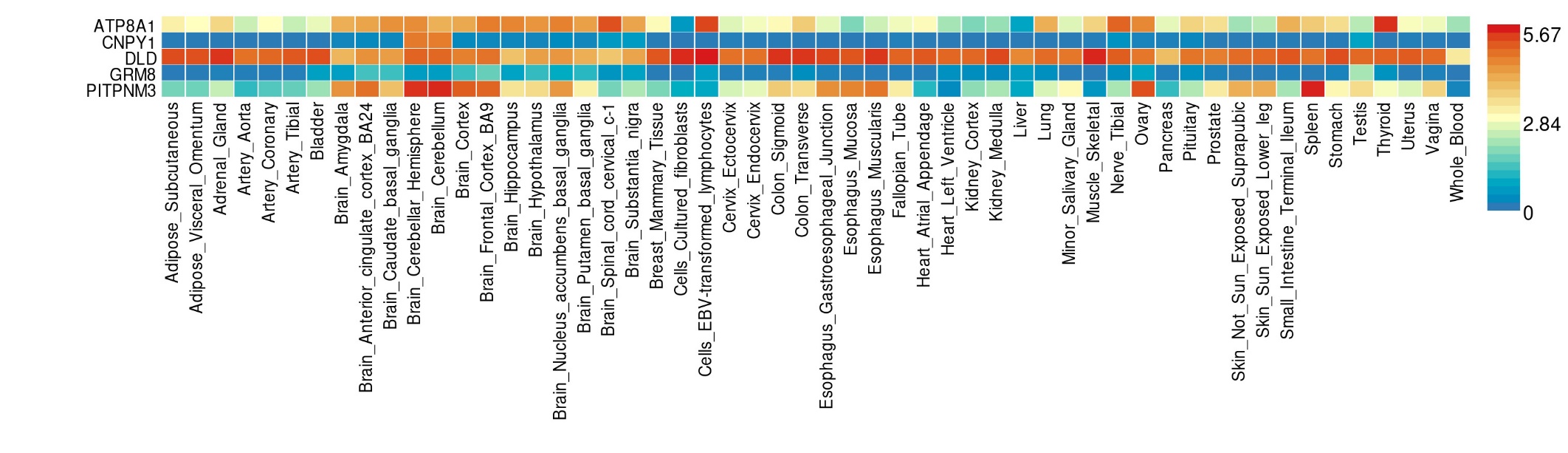


**Figure S2. Heatmap showing tissue based differential expression analysis using FUMA for genes mapped from genome-wide significant SNPs.**


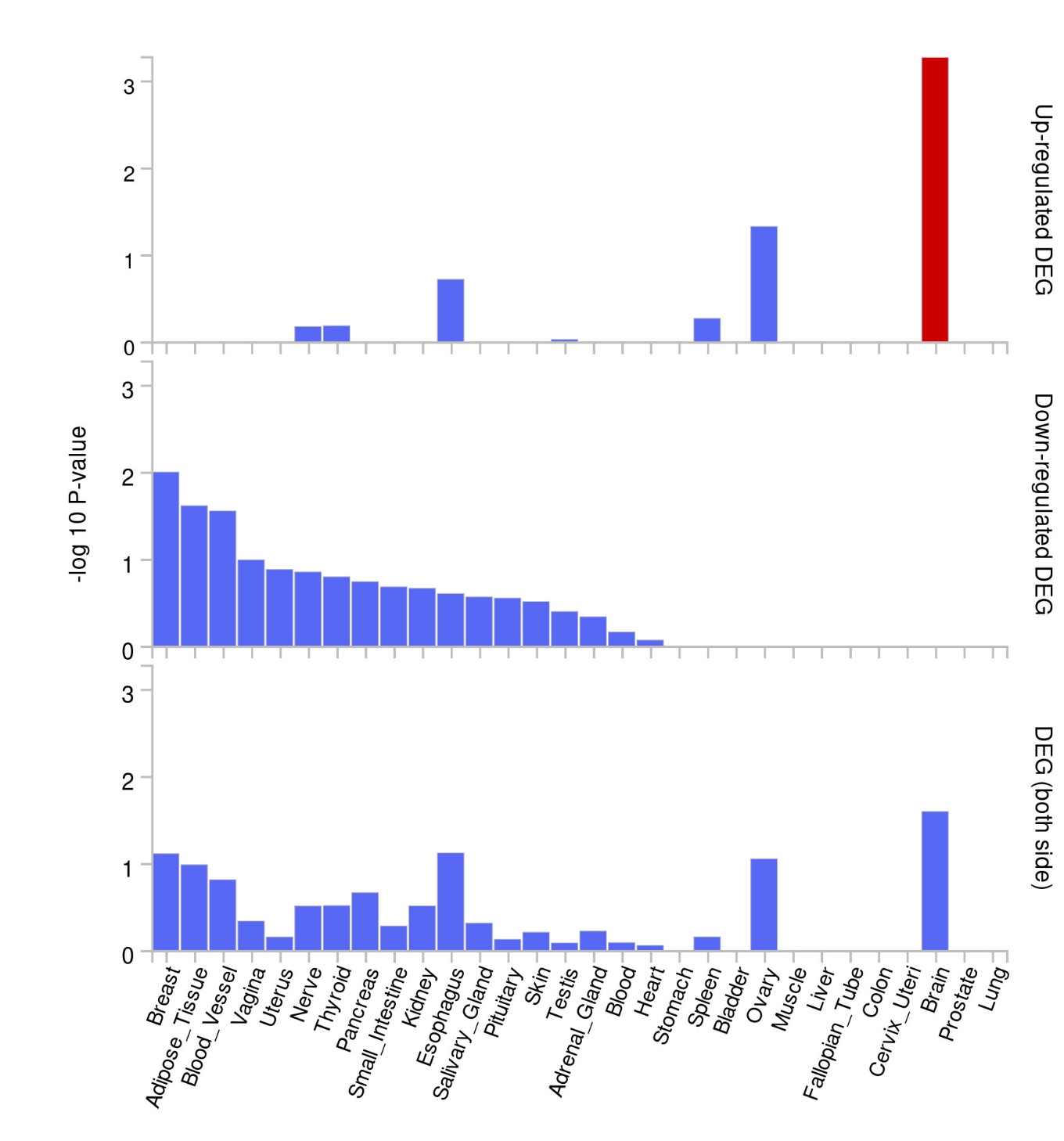


**Figure S3. General tissue specific up-regulated and down-regulated genes mapped from genome-wide significant SNPs via FUMA.**


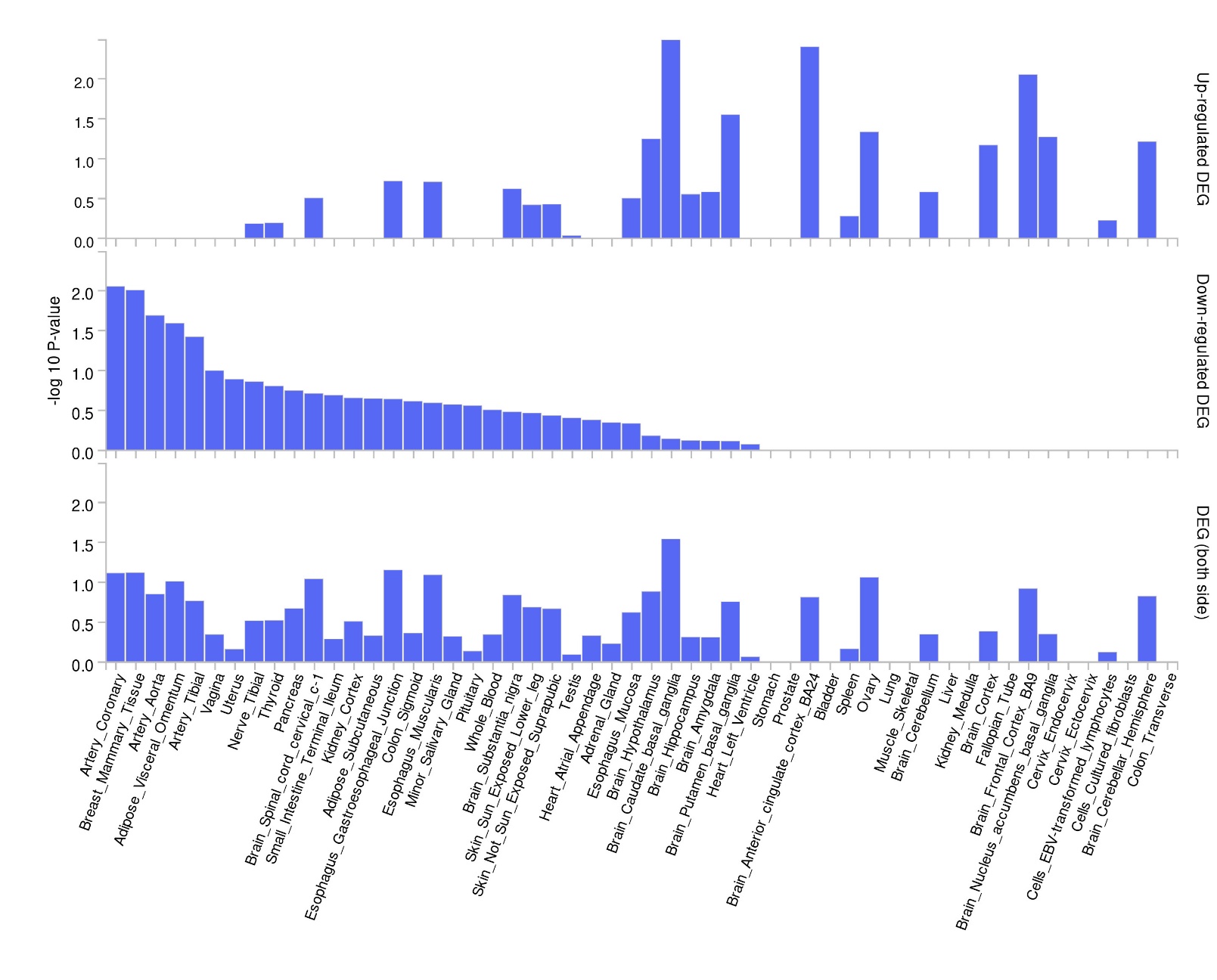


**Figure S4. Detailed tissue specific up-regulated and down-regulated genes mapped from genome-wide significant SNPs via FUMA**.

1. **Supplementary tables**

**Table S1. SNPs found previously to be associated with sepsis susceptibility in genome-wide association studies**

|  |  |  |  |  | **In this study** | | |  | **Previous study** | | |  |
| --- | --- | --- | --- | --- | --- | --- | --- | --- | --- | --- | --- | --- |
| SNP | CHR | Position | Minor allele | Major allele | MAF | HR | P-value | Gene | MAF | OR | P-value | **Reference** |
| rs7591064 | 2 | 48950462 | C | T | 0.05 | 1.57 | 0.02 | Intergenic | na | 0.67 | 9.50$\times$10^-6^ | Rautanen et al., 2015 |
| rs17324515 | 2 | 132638606 | T | C | 0.29 | 1.14 | 0.22 | *LYPD1* | na | 0.74 | 3.50$\times$10^-5^ |  |
| rs2709532 | 2 | 132668610 | G | C | 0.31 | 1.09 | 0.44 | *LYPD1* | na | 0.71 | 3.80$\times$10^-6^ |  |
| rs10928450 | 2 | 133328699 | G | A | 0.13 | 1.15 | 0.34 | *NCKAP5* | na | 0.63 | 9.00$\times$10^-6^ |  |
| rs13392963 | 2 | 133334077 | T | C | 0.12 | 1.13 | 0.42 | *NCKAP5* | na | 0.63 | 1.60$\times$10^-5^ |  |
| rs893357 | 2 | 200443763 | C | T | 0.15 | 0.9 | 0.45 | *SPATS2L* | na | 2.7 | 3.40$\times$10^-6^ |  |
| rs4957796 | 5 | 109066439 | C | T | 0.05 | 0.89 | 0.63 | *FER* | na | 0.52 | 9.70$\times$10^-8^ |  |
| rs975056 | 5 | 109070598 | C | T | 0.05 | 0.93 | 0.76 | *FER* | na | 0.56 | 3.30$\times$10^-7^ |  |
| rs62375529 | 5 | 109081631 | C | T | 0.05 | 0.96 | 0.85 | *FER* | na | 0.56 | 7.50$\times$10^-7^ |  |
| rs114618137 | 5 | 114052149 | C | T | 0.06 | 0.86 | 0.47 | lincRNA | na | 1.8 | 2.70$\times$10^-6^ |  |
| rs553438 | 5 | 135179038 | C | T | 0.21 | 1.1 | 0.45 | Intergenic | na | 0.63 | 2.20$\times$10^-5^ |  |
| rs639405 | 5 | 135196903 | A | T | 0.21 | 1.09 | 0.46 | Intergenic | na | 0.62 | 8.00$\times$10^-6^ |  |
| rs942635 | 6 | 163181229 | T | C | 0.23 | 1.07 | 0.54 | *PACRG* | na | 1.54 | 6.90$\times$10^-6^ |  |
| rs2763993 | 6 | 163182273 | T | C | 0.18 | 1.07 | 0.59 | *PACRG* | na | 1.49 | 4.60$\times$10^-5^ |  |
| rs4732529 | 7 | 84006270 | T | C | 0.35 | 0.97 | 0.78 | *SEMA3A* | na | 1.78 | 1.60$\times$10^-6^ |  |
| rs76881522 | 7 | 84040813 | A | T | 0.37 | 0.98 | 0.86 | *SEMA3A* | na | 1.77 | 6.20$\times$10^-6^ |  |
| rs35947027 | 7 | 123608684 | G | A | 0.40 | 1.09 | 0.39 | *ASB15* | na | 1.54 | 7.60$\times$10^-6^ |  |
| rs12114790 | 8 | 19697484 | C | T | 0.34 | 0.99 | 0.93 | *CSGALNACT1* | na | 1.43 | 6.40$\times$10^-5^ |  |
| rs10095344 | 8 | 19716732 | T | C | 0.33 | 1.01 | 0.94 | *CSGALNACT1* | na | 1.5 | 1.00$\times$10^-5^ |  |
| rs17057959 | 13 | 38395320 | C | T | 0.31 | 0.86 | 0.17 | Intergenic | na | 1.78 | 1.50$\times$10^-6^ |  |
| rs9566343 | 13 | 38528047 | G | T | 0.27 | 0.83 | 0.12 | Intergenic | na | 1.71 | 1.40$\times$10^-6^ |  |
| rs6501341 | 17 | 69669640 | G | A | 0.01 | 0.92 | 0.88 | *AC003051.1* | na | 1.98 | 3.50$\times$10^-6^ |  |
| rs2096460 | 21 | 32331791 | C | A | 0.12 | 0.94 | 0.71 | *URB1* | na | 0.61 | 8.10$\times$10^-6^ |  |
| rs382422 | 1 | 68450440 | C | G | 0.47 | 0.99 | 0.94 | *RPE65/*  *DEPDC1* | 0.22 | 2.1 | 3.21$\times$10^-6^ | Scherag et al., 2016 |
| rs72862231 | 3 | 37811568 | A | T | 0.08 | 0.89 | 0.54 | *ITGA9/*  *ITGA9-AS1* | 0.05 | 4.4 | 1.73$\times$10^-6^ |  |
| rs10933728 | 3 | 194309779 | G | A | 0.04 | 0.88 | 0.64 | *LINC00887* | 0.03 | 7.0 | 5.62$\times$10^-6^ |  |
| rs115550031 | 4 | 862314 | A | G | 0.03 | 0.92 | 0.80 | *GAK* | 0.02 | 13.8 | 2.45$\times$10^-6^ |  |
| rs945177 | 13 | 27047848 | A | G | 0.49 | 0.87 | 0.19 | *GPR12/USP12* | 0.02 | 14.7 | 1.31$\times$10^-6^ |  |
| rs2641697 | 16 | 84852171 | G | C | 0.19 | 1.13 | 0.33 | *CRISPLD2* | 0.36 | 2.0 | 5.99$\times$10^-6^ |  |
| rs7211184 | 17 | 14353766 | G | C | 0.06 | 0.9 | 0.63 | *HS3ST3B1/*  *CDRT7* | 0.72 | 2.0 | 9.43$\times$10^-6^ |  |
| rs12491812 | 3 | 50519150 | T | C | 0.31 | 0.93 | 0.51 | *CACNA2D2* | 0.011 | 7.32 | 4.18$\times$10^-11^ | Rosier et al., 2021 |
| rs2239753 | 3 | 50607727 | G | A | 0.32 | 0.87 | 0.21 | *CISH* | 0.011 | 7.02 | 2.80$\times$10^-11^ |  |
| rs2239752 | 3 | 50607982 | A | G | 0.32 | 0.87 | 0.21 | *CISH* | 0.011 | 5.62 | 5.43$\times$10^-10^ |  |
| rs2239751 | 3 | 50610457 | G | T | 0.32 | 0.87 | 0.21 | *CISH* | 0.011 | 5.62 | 5.21$\times$10^-10^ |  |
| rs743753 | 3 | 50613964 | T | C | 0.33 | 0.87 | 0.18 | *MAPKAPK3* | 0.011 | 5.62 | 5.21$\times$10^-10^ |  |
| rs616689 | 3 | 50631101 | T | C | 0.33 | 0.86 | 0.18 | *MAPKAPK3* | 0.014 | 5.79 | 1.87$\times$10^-10^ |  |
| rs9879397 | 3 | 50648211 | A | G | 0.33 | 0.86 | 0.17 | *MAPKAPK3* | 0.012 | 4.86 | 8.79$\times$10^-9^ |  |
| rs2170840 | 3 | 50649086 | C | A | 0.33 | 0.86 | 0.17 | *MAPKAPK3* | 0.014 | 5.78 | 1.87$\times$10^-10^ |  |
| rs12492982 | 3 | 50660724 | T | C | 0.33 | 0.87 | 0.20 | *MAPKAPK3* | 0.011 | 7.32 | 4.18$\times$10^-11^ |  |
| rs2035484 | 3 | 50684461 | C | T | 0.33 | 0.89 | 0.26 | *DOCK3* | 0.011 | 5.62 | 5.21$\times$10^-10^ |  |
| rs17051403 | 3 | 50714212 | A | C | 0.33 | 0.89 | 0.26 | *DOCK3* | 0.011 | 5.62 | 5.21$\times$10^-10^ |  |
| rs17072628 | 3 | 65244085 | A | G | 0.10 | 0.96 | 0.81 | . | 0.012 | 4.96 | 8.25$\times$10^-9^ |  |
| rs6763296 | 3 | 145991527 | C | T | 0.01 | 0.83 | 0.75 | . | 0.018 | 4.67 | 2.55$\times$10^-9^ |  |
| rs11991278 | 8 | 142919829 | T | C | 0.02 | 0.92 | 0.81 | *CYP11B2* | 0.01 | 6.87 | 8.48$\times$10^-9^ |  |
| rs6981918 | 8 | 142926523 | A | C | 0.02 | 0.92 | 0.81 | *CYP11B2* | 0.01 | 6.85 | 8.74$\times$10^-9^ |  |
| rs956727 | 9 | 84232018 | G | A | 0.23 | 0.86 | 0.20 | *SLC28A3* | 0.009 | 17.4 | 3.22$\times$10^-8^ |  |
| rs7953683 | 12 | 79599924 | T | C | 0.24 | 1.17 | 0.16 | *PAWR* | 0.024 | 2.07 | 3.07$\times$10^-8^ |  |
| rs10849640 | 12 | 119274332 | A | G | 0.20 | 1.22 | 0.09 | . | 0.116 | 2.25 | 3.22$\times$10^-8^ |  |
| rs10849641 | 12 | 119283549 | T | C | 0.20 | 1.17 | 0.19 | . | 0.115 | 2.27 | 2.65$\times$10^-8^ |  |
| rs10849642 | 12 | 119287509 | T | C | 0.20 | 1.14 | 0.27 | . | 0.117 | 2.25 | 4.04$\times$10^-8^ |  |
| rs11167801 | 5 | 143090769 | T | C | 0.44 | 1.09 | 0.41 | *ARHGAP26* | 0.09 | 0.13 | 8.77$\times$10^-8^ | D'Urso et al., 2020 |
| rs9489328 | 6 | 97656699 | T | G | 0.18 | 0.93 | 0.57 | *AL589740.1* | 0.1 | 0.1 | 1.05$\times$10^-10^ |  |
| rs368584 | 13 | 110492726 | G | C | 0.42 | 0.92 | 0.44 | *COL4A2* | 0.39 | 2.56 | 2.55$\times$10^-7^ |  |
| rs17128291 | 14 | 92416482 | G | A | 0.11 | 1.16 | 0.34 | Intergenic | 0.18 | 0.22 | 7.48$\times$10^-7^ |  |
| rs259492 | 3 | 21897935 | A | T | 0.48 | 1.13 | 0.22 | *ZNF385D* | 0.298 | 1.001 | 3.86$\times$10^-6^ | Neale et al., 2018 |
| rs34497103 | 4 | 79789170 | A | G | 0.32 | 0.99 | 0.95 | . | 0.155 | 1.001 | 4.08$\times$10^-6^ |  |
| rs36066750 | 4 | 79809381 | C | G | 0.39 | 1 | 0.97 | . | 0.156 | 1.001 | 4.95$\times$10^-6^ |  |
| rs7690505 | 4 | 79841481 | C | T | 0.32 | 1.02 | 0.85 | *PCAT4* | 0.155 | 1.001 | 1.64$\times$10^-6^ |  |
| rs4444770 | 4 | 79844900 | A | G | 0.32 | 1.05 | 0.67 | *LOC101927040* | 0.154 | 1.001 | 1.38$\times$10^-6^ |  |
| rs4690159 | 4 | 79860719 | C | G | 0.33 | 1.03 | 0.81 | *PCAT4* | 0.156 | 1.001 | 2.97$\times$10^-6^ |  |
| rs35981701 | 4 | 79869921 | T | C | 0.04 | 1.55 | 0.05 | . | 0.151 | 1.001 | 2.85$\times$10^-6^ |  |
| rs1466515 | 4 | 155593727 | A | G | 0.34 | 1.02 | 0.82 | . | 0.103 | 0.999 | 2.47$\times$10^-6^ |  |
| rs61406966 | 6 | 33081346 | A | G | 0.01 | 0.55 | 0.31 | *HLA-DPB1* | 0.012 | 1.003 | 5.13$\times$10^-6^ |  |
| rs16868789 | 6 | 33104821 | G | A | 0.01 | 0.55 | 0.31 | . | 0.014 | 1.003 | 5.23$\times$10^-6^ |  |
| rs79086515 | 7 | 78535620 | G | A | 0.07 | 0.97 | 0.88 | *DSCC1* | 0.015 | 1.003 | 1.24$\times$10^-6^ |  |
| rs74307801 | 9 | 114295648 | T | G | 0.41 | 0.98 | 0.82 | *COL27A1* | 0.059 | 0.998 | 2.89$\times$10^-6^ |  |
| rs4356266 | 11 | 111292165 | T | A | 0.38 | 0.93 | 0.48 | . | 0.295 | 1.001 | 5.73$\times$10^-6^ |  |
| rs7136342 | 12 | 91037279 | T | C | 0.21 | 1.01 | 0.95 | . | 0.176 | 0.999 | 5.70$\times$10^-6^ |  |
| rs61939273 | 12 | 130008690 | A | G | 0.02 | 1.29 | 0.44 | . | 0.111 | 1.001 | 1.67$\times$10^-6^ |  |
| rs75678135 | 14 | 80577277 | C | T | 0.17 | 0.99 | 0.93 | *CEP128* | 0.011 | 1.004 | 8.60$\times$10^-6^ |  |
| rs72740098 | 15 | 80239792 | G | A | 0.02 | 0.74 | 0.45 | *XND1* | 0.192 | 1.001 | 6.96$\times$10^-6^ |  |
| rs242323 | 21 | 27529230 | G | A | 0.20 | 0.98 | 0.90 | . | 0.147 | 1.001 | 3.81$\times$10^-6^ |  |
| rs28535971 | 22 | 17183406 | G | C | 0.05 | 0.87 | 0.56 | *ADA2* | 0.345 | 1.001 | 3.75$\times$10^-6^ |  |
| rs114658749 | 3 | 196048273 | T | C | 0.02 | 1.29 | 0.44 | *SDHAP1/*  *TFRC* | 0.062 | 1.44 | 5.56$\times$10^-6^ | Hernandez-Beeftink et al., 2022 |
| rs73285901 | 7 | 25150145 | A | G | 0.19 | 1.2 | 0.14 | *C7orf31* | 0.032 | 1.37 | 8.77$\times$10^-5^ |  |
| rs1573332 | 21 | 38530517 | A | T | 0.42 | 0.88 | 0.24 | *HS3ST3B1/*  *LOC101928475* | 0.345 | 1.33 | 1.14$\times$10^-5^ |  |

SNP: single nucleotide polymorphism, Chr: chromosome number, MAF: minor allele frequency, HR: hazard ratio, OR: odds ratio, na: not available. The SNPs reported from the previous study were identified through distinct databases and then combined; which were not made available (1). Blue rows denote the SNPs that were found atleast nominally significant in our study.

**Table S2. List of suggestively significant SNPs (*p*<1** **x10^-5^)**

|  |  |  |  |  |  |  |  |  |  |
| --- | --- | --- | --- | --- | --- | --- | --- | --- | --- |
| SNP | CHR | Position | Minor allele | Major allele | MAF | P-value | HR | Gene | MAF of 1KG EAS |
| rs61412352 | 1 | 36504220 | C | T | 0.2453 | 4.85$\times$10^-6^ | 1.638 | *.* | 0.2222 |
| rs80037391 | 1 | 169623536 | C | T | 0.0123 | 6.44$\times$10^-6^ | 4.011 | *SELP* | 0.0089 |
| rs141334171 | 1 | 169702520 | C | T | 0.018 | 9.75$\times$10^-6^ | 3.147 | *SELL* | 0.0109 |
| rs545094249 | 1 | 170061642 | T | A | 0.0161 | 3.72$\times$10^-6^ | 3.56 | *KIFAP3* | 0.0169 |
| rs191172722 | 1 | 181113732 | G | C | 0.0191 | 6.38$\times$10^-6^ | 3.132 | *.* | 0.0129 |
| rs76486211 | 2 | 2737108 | A | G | 0.0131 | 4.04$\times$10^-6^ | 3.158 | *.* | 0.0149 |
| rs1484678 | 2 | 22651000 | C | A | 0.417 | 4.60$\times$10^-6^ | 0.615 | *.* | 0.4494 |
| rs146295658 | 2 | 48579063 | T | A | 0.0101 | 6.62$\times$10^-7^ | 5.447 | *STON1* | 0.0119 |
| rs2052273 | 2 | 70717748 | A | G | 0.0168 | 4.79$\times$10^-6^ | 3.587 | *ADD2* | 0.0179 |
| rs61009552 | 2 | 98601021 | A | G | 0.0187 | 5.24$\times$10^-6^ | 3.255 | *COA5* | 0.0188 |
| rs77954059 | 2 | 105512911 | T | C | 0.0512 | 6.00$\times$10^-6^ | 2.200 | *.* | 0.0427 |
| rs139973594 | 2 | 111084233 | C | G | 0.0131 | 1.25$\times$10^-6^ | 4.018 | *ACOXL* | 0.0139 |
| rs555535158 | 2 | 127674034 | G | C | 0.0109 | 6.00$\times$10^-6^ | 3.835 | *LIMS2* | 0.0099 |
| rs146434516 | 2 | 165795651 | A | T | 0.0109 | 6.43$\times$10^-6^ | 3.980 | *GALNT3* | 0.002 |
| rs12615695 | 2 | 211105039 | C | G | 0.3295 | 7.66$\times$10^-6^ | 1.606 | *.* | 0.369 |
| rs138054007 | 3 | 188123354 | C | A | 0.0146 | 7.28$\times$10^-7^ | 4.264 | *.* | 0.0129 |
| rs75123867 | 3 | 191375496 | T | G | 0.0236 | 5.43$\times$10^-6^ | 2.909 | *CCDC50* | 0.0248 |
| rs150285457 | 4 | 42407286 | A | AG | 0.0165 | 2.67$\times$10^-6^ | 3.400 | *.* | 0.006 |
| rs189940971 | 4 | 42574493 | T | C | 0.0116 | 1.54$\times$10^-8^ | 4.862 | *ATP8A1* | 0.0079 |
| rs150193664 | 4 | 64028172 | G | T | 0.0105 | 3.19$\times$10^-6^ | 4.724 | *.* | 0.001 |
| rs1262422195 | 4 | 64462367 | T | TG | 0.0101 | 2.19$\times$10^-6^ | 4.863 | *.* | 0.0009 |
| rs143415165 | 4 | 156020684 | A | G | 0.0161 | 1.27$\times$10^-6^ | 3.326 | *.* | 0.0248 |
| rs141848774 | 4 | 164561575 | T | C | 0.0262 | 8.03$\times$10^-6^ | 2.688 |  | 0.0367 |
| rs141687698 | 4 | 171581773 | G | T | 0.0303 | 7.67$\times$10^-7^ | 2.908 | *LOC105377535* | 0.0387 |
| rs190492532 | 4 | 174386231 | T | C | 0.0116 | 1.59$\times$10^-7^ | 4.819 | *LOC105377547* | 0.006 |
| rs145571907 | 5 | 9186829 | C | G | 0.0112 | 3.69$\times$10^-6^ | 4.151 | *SEMA5A* | 0.0149 |
| rs141398409 | 5 | 85164410 | A | G | 0.0389 | 2.18$\times$10^-6^ | 2.421 | *.* | 0.0308 |
| rs57889678 | 5 | 107515464 | T | C | 0.0195 | 7.44$\times$10^-6^ | 3.019 | *EFNA5* | 0.0079 |
| rs59488533 | 5 | 149017638 | A | G | 0.0554 | 3.37$\times$10^-6^ | 2.236 | *SH3TC2* | 0.0516 |
| rs180783760 | 5 | 161410265 | A | G | 0.0142 | 6.19$\times$10^-6^ | 3.606 | *GABRB2* | 0.0079 |
| rs529185079 | 6 | 111243144 | G | GGA | 0.0105 | 2.66$\times$10^-7^ | 4.850 | *MFSD4B-DT* | 0.0129 |
| rs148846953 | 6 | 164059503 | T | A | 0.0176 | 2.10$\times$10^-6^ | 3.710 | *.* | 0.0069 |
| rs73135595 | 7 | 78776093 | C | T | 0.0101 | 1.58$\times$10^-7^ | 6.165 | *MAGI2* | 0.0069 |
| rs141257999 | 7 | 107896660 | C | T | 0.0127 | 3.89$\times$10^-8^ | 4.453 | *DLD* | 0.0159 |
| rs1419466 | 7 | 126921264 | T | C | 0.0295 | 3.47$\times$10^-10^ | 3.836 | *GRM8* | 0.0347 |
| rs11769197 | 7 | 155542369 | C | G | 0.0123 | 3.89$\times$10^-8^ | 4.562 | *CNPY1* | 0.0238 |
| rs58643122 | 7 | 157911234 | A | C | 0.0176 | 1.75$\times$10^-6^ | 3.123 | *PTPRN2* | 0.0129 |
| rs202027642 | 7 | 158131064 | A | AAC | 0.0116 | 4.58$\times$10^-6^ | 4.795 | *PTPRN2* | 0.3026 |
| rs141157595 | 8 | 75086786 | G | A | 0.0112 | 6.53$\times$10^-7^ | 4.373 | *.* | 0.0188 |
| rs192047328 | 8 | 109671690 | G | T | 0.0127 | 6.27$\times$10^-7^ | 3.974 | *SYBU* | 0.0179 |
| rs556879416 | 8 | 111656682 | AG | A | 0.0146 | 4.76$\times$10^-7^ | 3.892 | *.* | 0.0099 |
| rs10219138 | 10 | 17558128 | C | A | 0.0172 | 5.70$\times$10^-7^ | 3.674 | *.* | 0.0159 |
| rs1056103640 | 10 | 17914683 | C | T | 0.0112 | 1.84$\times$10^-6^ | 4.330 | *.* | 0.0094 |
| rs117082556 | 10 | 30161539 | C | A | 0.0613 | 4.48$\times$10^-6^ | 2.040 | *.* | 0.0516 |
| rs149165514 | 10 | 51974143 | A | T | 0.0116 | 4.58$\times$10^-6^ | 3.935 | *PRKG1* | 0.0139 |
| rs142973266 | 10 | 55350831 | G | A | 0.0273 | 8.64$\times$10^-6^ | 2.708 | *PCDH15* | 0.0208 |
| rs190194973 | 10 | 63140516 | G | T | 0.015 | 2.55$\times$10^-6^ | 3.837 | *NRBF2* | 0.0139 |
| rs2660075 | 10 | 64382040 | T | C | 0.0363 | 6.27$\times$10^-6^ | 2.456 | *LOC124902439* | 0.0625 |
| rs11198664 | 10 | 118891269 | T | C | 0.0352 | 5.07$\times$10^-7^ | 2.862 | *.* | 0.0298 |
| rs138219621 | 11 | 8766557 | T | C | 0.0142 | 4.59$\times$10^-7^ | 3.505 | *DENND2B* | 0.0109 |
| rs186289726 | 11 | 97211337 | A | C | 0.0236 | 2.11$\times$10^-6^ | 3.010 | *.* | 0.0238 |
| rs61909781 | 11 | 100273351 | C | G | 0.0325 | 1.11$\times$10^-7^ | 2.929 | *CNTN5* | 0.0565 |
| rs148910068 | 11 | 117110595 | T | C | 0.0202 | 4.88$\times$10^-6^ | 3.162 | *.* | 0.0248 |
| rs376211160 | 12 | 68129005 | G | A | 0.0168 | 6.63$\times$10^-6^ | 3.551 |  | 0.0069 |
| rs184756283 | 13 | 62476531 | A | G | 0.0105 | 2.26$\times$10^-6^ | 4.367 | *.* | 0.004 |
| rs79252572 | 13 | 67870848 | A | AC | 0.0378 | 1.69$\times$10^-6^ | 2.451 | *.* | 0.0437 |
| rs140736840 | 13 | 79287080 | A | G | 0.0153 | 7.78$\times$10^-7^ | 3.466 | *.* | 0.0089 |
| rs370716825 | 13 | 79581814 | A | C | 0.0161 | 8.76$\times$10^-7^ | 3.452 | *.* | 0.0119 |
| rs75766683 | 13 | 88643260 | T | C | 0.0195 | 1.90$\times$10^-6^ | 3.174 | *.* | 0.0298 |
| rs74570040 | 13 | 101154222 | G | A | 0.0468 | 2.89$\times$10^-6^ | 2.221 | *NALCN* | 0.0407 |
| rs961137 | 14 | 77987341 | G | A | 0.0359 | 4.17$\times$10^-6^ | 2.479 | *.* | 0.0655 |
| rs4381519 | 14 | 78047731 | T | C | 0.0127 | 2.72x10^-6^ | 3.687 | *.* | 0.0367 |
| rs77502443 | 14 | 96565910 | G | C | 0.0213 | 5.96$\times$10^-6^ | 2.804 | *PAPOLA* | 0.0139 |
| rs1992236 | 15 | 55588913 | A | G | 0.012 | 2.76$\times$10^-6^ | 3.777 | *PYGO1* | 0.0109 |
| rs552244933 | 15 | 88288537 | GA | G | 0.0153 | 7.46$\times$10^-6^ | 3.472 | *.* | 0.0169 |
| rs9931031 | 16 | 492289 | C | T | 0.2341 | 5.66$\times$10^-6^ | 1.659 | *RAB11FIP3* | 0.2609 |
| rs76818150 | 16 | 10955006 | T | C | 0.1331 | 6.74$\times$10^-7^ | 1.904 | *CLEC16A* | 0.13 |
| rs118123390 | 16 | 24265267 | A | G | 0.0228 | 5.95$\times$10^-8^ | 3.194 | *CACNG3* | 0.0129 |
| rs75021908 | 16 | 85156869 | T | C | 0.0587 | 3.10$\times$10^-6^ | 2.229 | *LOC105371382* | 0.0526 |
| rs139662071 | 16 | 85199888 | A | G | 0.0411 | 1.76$\times$10^-6^ | 2.601 | *GSE1* | 0.0367 |
| rs117656134 | 16 | 87034857 | T | C | 0.0142 | 1.28$\times$10^-6^ | 3.777 | *.* | 0.0169 |
| rs140453155 | 17 | 6463959 | A | G | 0.0127 | 4.64$\times$10^-8^ | 4.525 | *PITPNM3* | 0.0149 |
| rs141878780 | 17 | 11581488 | C | G | 0.0146 | 3.43$\times$10^-6^ | 3.547 | *.* | 0.0159 |
| rs75112108 | 17 | 19338445 | A | C | 0.0191 | 8.62$\times$10^-6^ | 3.258 | *B9D1* | 0.0288 |
| rs146520211 | 17 | 48851677 | A | C | 0.0277 | 1.85$\times$10^-6^ | 2.612 | *CALCOCO2* | 0.0218 |
| rs9905428 | 17 | 48871347 | G | C | 0.1657 | 5.28$\times$10^-6^ | 1.735 | *.* | 0.1786 |
| rs80070296 | 17 | 64850601 | A | G | 0.0247 | 2.40$\times$10^-6^ | 2.801 | *LOC105376844* | 0.0347 |
| rs940034052 | 18 | 49409284 | A | AAAAAAAAAAAAAAAAG | 0.0168 | 2.40$\times$10^-6^ | 3.298 | *DYM* | no data |
| rs138873663 | 18 | 68953655 | A | G | 0.0116 | 3.48$\times$10^-6^ | 4.238 | *CCDC102B* | 0.0129 |
| rs143510397 | 19 | 52568491 | G | GGCATCATTGT | 0.046 | 7.62$\times$10^-6^ | 2.305 | *ZNF808/ ZNF701* | 0.0417 |
| rs13045660 | 20 | 574840 | A | G | 0.368 | 4.01$\times$10^-6^ | 1.600 | *.* | 0.3442 |
| rs143210611 | 20 | 580911 | CA | C | 0.1096 | 2.18$\times$10^-7^ | 1.989 | *.* | 0.1239 |
| rs79733107 | 20 | 40578757 | G | C | 0.0105 | 4.50$\times$10^-6^ | 4.001 | *LOC102724968* | 0.0119 |
| rs6065709 | 20 | 44198646 | T | A | 0.0344 | 1.14$\times$10^-6^ | 2.447 | *OSER1* | 0.0278 |
| rs182546191 | 21 | 19288500 | A | C | 0.012 | 4.58$\times$10^-6^ | 4.460 | *.* | 0.002 |
| rs181165672 | 22 | 26866534 | T | C | 0.0131 | 2.91$\times$10^-6^ | 4.007 | *LOC110091768* | 0.006 |

SNP: single nucleotide polymorphism, Chr: chromosome number, MAF: minor allele frequency, HR: hazard ratio, 1KG: 1000 genomes, EAS: East Asians

**Table S3.** **The performance of the polygenic risk score in the Cox prediction model using clumping and thresholding method (C+T) parameters**

|  | |  | PRS-based prediction model^+^ | | | | |
| --- | --- | --- | --- | --- | --- | --- | --- |
| r^2^ | p-value | number of SNPs | C-index | SE (C-index) | HR | 95% CI | p-value |
| **0.2** | 1$\times$10^-5^ | 86 | 0.68 | 0.04 | 1.57 | (1.16 – 2.12) | 0.003* |
|  | 1$\times$10^-4^ | 395 | 0.67 | 0.05 | 1.94 | (1.37 – 2.74) | <0.001* |
|  | 1$\times$10^-3^ | 2215 | 0.71 | 0.04 | 2.04 | (1.38 – 3.01) | <0.001* |
|  | 0.01 | 13055 | 0.77 | 0.04 | 3.10 | (1.74– 5.52) | <0.001* |
|  | **0.05** | **48546** | **0.78** | **0.04** | **5.62** | **(2.43– 13.01)** | <0.001***** |
| 0.4 | 1$\times$10^-5^ | 87 | 0.68 | 0.04 | 1.61 | (1.18 – 2.19) | 0.003* |
|  | 1$\times$10^-4^ | 407 | 0.70 | 0.05 | 2.09 | (1.43 – 3.07) | <0.001* |
|  | 1$\times$10^-3^ | 2365 | 0. 70 | 0.05 | 1.94 | (1.33 – 2.83) | <0.001* |
|  | 0.01 | 14820 | 0.75 | 0.04 | 2.76 | (1.62 – 4.71) | <0.001* |
|  | 0.05 | 58960 | 0.7633 | 0.04 | 4.54 | (2.13 – 9.68) | <0.001* |
| 0.6 | 1$\times$10^-5^ | 88 | 0.68 | 0.04 | 1.65 | (1.20 – 2.26) | 0.002* |
|  | 1$\times$10^-4^ | 433 | 0.70 | 0.05 | 2.10 | (1.45 – 3.03) | <0.001* |
|  | 1$\times$10^-3^ | 2598 | 0.70 | 0.05 | 1.88 | (1.31 – 2.69) | <0.001* |
|  | 0.01 | 16912 | 0.74 | 0.04 | 2.46 | (1.52 – 3.98) | <0.001* |
|  | 0.05 | 70230 | 0.76 | 0.04 | 3.55 | (1.85 – 6.82) | <0.001* |
| 0.8 | 1$\times$10^-5^ | 95 | 0.68 | 0.04 | 1.64 | (1.21 – 2.21) | 0.001* |
|  | 1$\times$10^-4^ | 483 | 0.68 | 0.05 | 2.08 | (1.41 – 3.06) | <0.001* |
|  | 1$\times$10^-3^ | 2996 | 0.70 | 0.05 | 1.86 | (1.31 – 2.66) | <0.001* |
|  | 0.01 | 20299 | 0.72 | 0.05 | 2.14 | (1.39 – 3.30) | <0.001* |
|  | 0.05 | 87380 | 0.76 | 0.04 | 2.97 | (1.70 – 5.19) | <0.001* |

CI: confidence interval, HR: hazard ratio, PRS: polygenic risk score, SE: standard error, SNP: single nucleotide polymorphism. Bold: depicts the C+T threshold chosen for PRS-sepsis1, PRS-sepsis2 and PRS-sepsis3, ^+^ Models were adjusted by age, sex, and the first three principal components, * p < 0.05

**Table S4. Characteristics associated with 28-day mortality in the training dataset.**

| Characteristics | Missing data | 28-day Survivor (n=1091) | Death  (n=166) | P-value |
| --- | --- | --- | --- | --- |
| Age | 0 (0) | 61.5 (15.4) | 70.7 (13.7) | <0.001 |
| Sex (Female), *n (%)* | 0 (0) | 488 (44.7) | 63 (38.0) | 0.12 |
| Origin from ED, *n (%)* | 0 (0) | 936 (85.8) | 153 (92.2) | 0.0335 |
| Length of ED stay, *hour* | 0 (0) | 20.8 (23.5) | 14.1 (14.3) | 0.001 |
| Septic shock, *n (%)* |  |  |  |  |
| In-hospital | 0 (0) | 465 (42.6) | 115 (69.3) | <0.001 |
| In-hospital but not ED | 0 (0) | 357 (32.7) | 104 (62.7) | <0.001 |
| ED | 0 (0) | 199 (18.2) | 48 (28.9) | 0.002 |
| ***Severity*** |  |  |  |  |
| CCI score | 0 (0) | 4.9 (3.3) | 7.7 (3.2) | <0.001 |
| Total SOFA score | 0 (0) | 7.4 (3.0) | 8.3 (2.9) | 0.001 |
| SOFA score of platelets | 0 (0) | 0.5 (0.9) | 0.8 (1.1) | 0.01 |
| SOFA score of creatinine | 0 (0) | 1.1 (1.3) | 1.8 (1.4) | <0.001 |
| SOFA score of circulatory | 0 (0) | 1.1 (1.6) | 0.8 (1.4) | 0.49 |
| SOFA score of bilirubin | 0 (0) | 0.5 (0.9) | 0.7 (1.1) | 0.39 |
| ***Laboratory tests*** |  |  |  |  |
| C-reactive protein | 115 (9) | 12.5 (10.7) | 13.6 (10.8) | 0.17 |
| Procalcitonin | 607 (48) | 15.8 (29.0) | 17.6 (27.1) | 0.002 |
| NLR | 222 (18) | 15.9 (20.3) | 19.0 (21.6) | <0.001 |
| ***Treatments*** |  |  |  |  |
| Norepinephrine usage, *n (%)* |  |  |  |  |
| In-hospital | 0 (0) | 411 (37.7) | 121 (72.9) | <0.001 |
| In-hospital but not ED | 0 (0) | 306 (28.0) | 104 (62.7) | <0.001 |
| ED | 0 (0) | 232 (21.3) | 60 (36.1) | <0.001 |

**Table S5. Characteristics associated with 28-day mortality in the testing dataset.**

| Characteristics | Missing data | 28-day Survivor (n=40) | Death  (n=40) | P-value |
| --- | --- | --- | --- | --- |
| Age | 0 (0) | 59.6 (16.2) | 73.4 (13.1) | <0.001 |
| Sex (Female), *n (%)* | 0 (0) | 13 (32.5) | 13 (32.5) | 1.0 |
| Origin from ED, *n (%)* | 0 (0) | 35 (87.5) | 35 (87.5) | 1.0 |
| Length of ED stay, *hour* | 0 (0) | 18.3 (21.5) | 13.3 (11.8) | 0.76 |
| Septic shock, *n (%)* |  |  |  |  |
| In-hospital | 0 (0) | 19 (47.5) | 31 (77.5) | 0.01 |
| In-hospital but not ED | 0 (0) | 14 (35.0) | 27 (67.5) | 0.007 |
| ED | 0 (0) | 10 (25.0) | 12 (30.0) | 0.80 |
| ***Severity*** |  |  |  |  |
| CCI score | 0 (0) | 3.2 (3.0) | 7.7 (3.6) | <0.001 |
| Total SOFA score | 0 (0) | 7.2 (2.5) | 8.7 (3.6) | 0.06 |
| SOFA score of platelets | 0 (0) | 0.3 (0.6) | 0.8 (1.1) | 0.01 |
| SOFA score of creatinine | 0 (0) | 0.8 (1.0) | 1.8 (1.3) | <0.001 |
| SOFA score of circulatory | 0 (0) | 1.6 (1.7) | 0.8 (1.5) | 0.01 |
| SOFA score of bilirubin | 0 (0) | 0.4 (0.7) | 0.8 (1.2) | 0.27 |
| ***Laboratory tests*** |  |  |  |  |
| C-reactive protein | 3 (4) | 14.5 (11.8) | 13.8 (11.2) | 0.94 |
| Procalcitonin | 29 (36) | 12.6 (30.2) | 9.7 (19.2) | 0.03 |
| NLR | 19 (24) | 20.0 (34.8) | 24.3 (28.6) | <0.001 |
| ***Treatments*** |  |  |  |  |
| Norepinephrine usage, *n (%)* |  |  |  |  |
| In-hospital | 0 (0) | 15 (37.5) | 30 (75.0) | 0.002 |
| In-hospital but not ED | 0 (0) | 12 (30.0) | 26 (65.0) | 0.004 |
| ED | 0 (0) | 8 (20.0) | 15 (37.5) | 0.14 |

References

1. Rautanen A, Mills TC, Gordon AC, Hutton P, Steffens M, Nuamah R, et al. Genome-wide association study of survival from sepsis due to pneumonia: an observational cohort study. The Lancet Respiratory Medicine. 2015;3(1):53-60.
